# The E3 ubiquitin ligase RNF40 suppresses apoptosis in colorectal cancer cells

**DOI:** 10.1186/s13148-019-0698-x

**Published:** 2019-07-02

**Authors:** Deborah Schneider, Robert Lorenz Chua, Nicole Molitor, Feda H. Hamdan, Eva Maria Rettenmeier, Evangelos Prokakis, Vivek Kumar Mishra, Vijayalakshmi Kari, Florian Wegwitz, Steven A. Johnsen, Robyn Laura Kosinsky

**Affiliations:** 1 Department of General, Visceral and Pediatric Surgery, University Medical Center Göttingen, Justus-von-Liebig-Weg 11, 37077 Göttingen, Germany; 2 Gene Regulatory Mechanisms and Molecular Epigenetics Lab, Division of Gastroenterology and Hepatology, Mayo Clinic, 200 First St SW, Rochester, MN USA; 3 Department of Dermatology and the Helen Diller Family Comprehensive Cancer Center, University of California, San Francisco, San Francisco, CA USA

**Keywords:** Colorectal cancer, Apoptosis, RNF40, H2Bub1, Caspases

## Abstract

**Background:**

Colorectal cancer (CRC) is the fourth leading cause of cancer-related deaths worldwide, and deciphering underlying molecular mechanism is essential. The loss of monoubiquitinated histone H2B (H2Bub1) was correlated with poor prognosis of CRC patients and, accordingly, H2Bub1 was suggested as a tumor-suppressive mark. Surprisingly, our previous work revealed that the H2B ubiquitin ligase RING finger protein 40 (RNF40) might exert tumor-promoting functions. Here, we investigated the effect of RNF40 loss on tumorigenic features of CRC cells and their survival in vitro.

**Methods:**

We evaluated the effects of RNF40 depletion in several human CRC cell lines in vitro. To evaluate cell cycle progression, cells were stained with propidium iodide and analyzed by flow cytometry. In addition, to assess apoptosis rates, caspase 3/7 activity was assessed in a Celigo® S-based measurement and, additionally, an Annexin V assay was performed. Genomic occupancy of H2Bub1, H3K79me3, and H3K27ac was determined by chromatin immunoprecipitation. Transcriptome-wide effects of RNF40 loss were evaluated based on mRNA-seq results, qRT-PCR, and Western blot. To rescue apoptosis-related effects, cells were treated with Z-VAD-FMK.

**Results:**

Human CRC cell lines displayed decreased cell numbers in vitro after RNF40 depletion. While the differences in confluence were not mediated by changes in cell cycle progression, we discovered highly increased apoptosis rates after RNF40 knockdown due to elevated caspase 3/7 activity. This effect can be explained by reduced mRNA levels of anti-apoptotic and upregulation of pro-apoptotic BCL2 family members. Moreover, the direct occupancy of the RNF40-mediated H2B monoubiquitination was observed in the transcribed region of anti-apoptotic genes. Caspase inhibition by Z-VAD-FMK treatment rescued apoptosis in RNF40-depleted cells. However, knockdown cells still displayed decreased tumorigenic features despite the absence of apoptosis.

**Conclusions:**

Our findings reveal that RNF40 is essential for maintaining tumorigenic features of CRC cells in vitro by controlling the expression of genes encoding central apoptotic regulators.

**Electronic supplementary material:**

The online version of this article (10.1186/s13148-019-0698-x) contains supplementary material, which is available to authorized users.

## Background

Colorectal cancer (CRC) is one of the major causes of cancer-related deaths and incidence rates among people younger than 50 years have risen [1]. Generally, the formation of CRC is a multistep process initiated by a hyperproliferation of the intestinal epithelium, leading to the formation of pre-cancerous polyps and, finally, adenomas and adenocarcinomas [2]. This transition is based on the accumulation of molecular alterations, which are associated with the activation of oncogenes and the inactivation of tumor suppressor genes [3]. These (epi-)genetic changes allow the cells to adopt characteristics referred to as “hallmarks of cancer” such as self-sufficiency in growth signals, limitless proliferative potential, and evasion of apoptosis [4].

The regulation of apoptosis, programmed cell death, is a tightly controlled process under physiological and pathological conditions and is essential for the clearance of cells displaying irreparable damage. The BCL-2 protein family plays a key role in controlling cell death and is subdivided into the pro-apoptotic (e.g., BAD, BAK, BAX, BCL-xS, BIM, and HRK) and anti-apoptotic factors (e.g., BCL-2, BCL-xL, and MCL1) [5]. These proteins control cytochrome-c release from the mitochondria, which results in the activation of a cascade of cysteine-aspartic proteases (caspases). Caspases mediate the cleavage of a broad variety of proteins responsible for normal cellular functions as well as cellular structure [6, 7]. As a consequence, a distinct morphological appearance, membrane blebbing, can be observed and the cell undergoes cell death [8]. Since the evasion of apoptosis is a characteristic hallmark of cancer cells, induced cell death could cause the clearance of malignant cells and, therefore, presents a mechanism of targeting cancer cells [9]. As reviewed by Baig and colleagues [10], several therapeutic approaches and agents are currently being tested clinically, including BH3 mimetics, which target proteins of the BCL-2 family in order to trigger apoptosis.

Interestingly, knockdown of the RING finger protein RNF20 was shown to downregulate the anti-apoptotic factor *BCL-2* at the mRNA level in vitro [11]. Generally, RNF20 and RNF40 form an obligate heterodimer with RNF40 which is recruited by the adaptor protein WW domain-containing adapter protein with coiled-coil (WAC) protein to the elongating RNA polymerase II large subunit following phosphorylation of serine 2 of the C-terminal heptapeptide repeat sequence [12]. By exerting its E3 ligase activity, the RNF20/RNF40 complex was shown to monoubiquitinate histone H2B at lysine 120 (H2Bub1). It was proposed that H2Bub1 is associated with active transcriptional elongation by promoting the recruitment of the facilitates chromatin transcription (FACT) complex, which enhances chromatin accessibility and eases the passage of RNA polymerase II through the chromatin across the gene body [13]. Importantly, H2Bub1 was described as a tumor-suppressive mark since reduced levels were associated with advanced tumor grade and poor survival in colorectal cancer patients [14]. By extension, it has been postulated that the E3 ligases mediating the monoubiquitination of H2B also have a tumor suppressive function. Intriguingly, we previously demonstrated that the transient loss of RNF40 and accompanying the loss of H2Bub1 resulted in reduced proliferative potential of several CRC cell lines in vitro [15].

In this study, we used multiple approaches to investigate the mechanisms underlying these effects and have identified a previously unknown role for RNF40 and H2Bub1 in maintaining the expression of several anti-apoptotic genes. Together, these findings suggest that RNF40-mediated H2B monoubiquitination has a highly context-dependent function and may exert pro-tumorigenic functions in certain cellular contexts and thereby serve as a potential anti-cancer target.

## Methods

### Cell culture

Human colorectal cancer cell lines were grown in growth medium (HCT116, HT-29: McCoy’s; RKO, SW48, SW837: Dulbecco's Modified Eagle's Medium/F12) supplemented with 10% fetal bovine serum, 100 units/ml penicillin, and 100 μg/ml streptomycin at 37 °C and 5% CO_2_. siRNA (GE Dharmacon siGENOME; non-targeting siRNA 5 [D-001210-05-20], RNF40 siRNAs [D-006913-01, -02, -03, -04]) transfections were performed using Lipofectamine® RNAiMAX (Invitrogen) according to the manufacturer’s instructions. Twenty-four hours after siRNA transfection, cells were treated with 80 μM Z-VAD-FMK (Adooq) dissolved in DMSO or DMSO alone as a negative control for 48 h.

### CRISPR/Cas9-mediated deletion of *RNF40*

pSpCas9(BB)-2A-GFP (PX458) was a gift from Feng Zhang (Addgene plasmid #48138) [16] and was used to transfect cells with Cas9 along with the targeting guide RNAs (gRNAs). Guide RNAs were designed and checked for efficiency (http://cistrome.org/SSC) and specificity (http://crispr.mit.edu). Subsequently, they were cloned in the plasmid and transfected into cells using the Lonza Nucleofector 2b device according to manufacturer’s instructions (Kit T, program O-017). After 48 h of transfection, cells were sorted by flow cytometry (Cell Sorting Core Facility, Department of Hematology and Medical Oncology, University Medical Center Göttingen) and cells with highest GFP positivity were transferred as single cells into 96-well plates and propagated. For initial validation, cells were transfected with Lipofectamine 2000® (Life Technologies) according to the manufacturer’s instructions. gRNA sequences are listed in Additional file 3: Table S1.

### Validation of CRISPR/Cas9-mediated *RNF40* knockout by PCR

DNA was extracted from cells grown on 6-well plates by adding 300 μl lysis buffer (0.2% SDS, 100 mM Tris-HCl pH 8.5, 5 mM EDTA, 200 mM NaCl) and 40 μg proteinase K with incubation at 56 °C and shaking overnight. DNA was precipitated with isopropanol and washed with 70% ethanol twice and re-constituted in water. DNA (100 ng) was amplified by PCR with 0.4 U Phusion polymerase (Thermo Scientific), 1× high fidelity buffer, 0.2 mM dNTPs, and 1 μM forward and reverse primers. The samples were heated at 98 °C for 3 min followed by 35 cycles of 98 °C for 30 s, 60 °C for 30 s, and 72 °C for 60 s. Finally, extension was performed for 10 min at 72 °C. Forward primer: 5′-AGAAGCTCAGAACACGACGC-3′, reverse primer: 5′-TGCGTATCACATCCTCAGGG-3′. A PCR product of 1168 base pairs was expected in *RNF40* wild-type cells and 312 bp in *RNF40* knockout cells.

### Validation of CRISPR/Cas9-mediated *RNF40* knockout by immunofluorescence

Cells were grown on glass cover slips in 24-well plates for 24 h, and then washed three times with PBS and fixed using 4% paraformaldehyde in PBS for 10 min. Subsequently, cells were permeabilized with 0.5% Triton X-100 for 10 min and blocked with 3% BSA for 30 min prior to overnight incubation with RNF40 antibody (Sigma Aldrich, R9029) at 4 °C. Cells were incubated for 1 h in secondary antibody conjugated to Alexa® Flour 594 (Life Technologies). Nuclei were stained with DAPI and coverslips were mounted on glass slides and left to dry at room temperature for 1 h and then stored at 4 °C. Pictures were taken with a Zeiss LSM 510 Meta confocal 258 microscope.

### Cell characterization assays

Characterization assays were performed as previously described [15]. Briefly, cells were seeded 24 h post siRNA transfection. Proliferation was tested by seeding 2000–5000 cells onto 96-well assay plates (Corning Life Sciences) and measuring the confluence daily using a Celigo® S cell imaging cytometer (Nexcelom Bioscience LLC). Clonogenic growth was assessed by seeding 500 cells onto a 6-well plate and waiting until macroscopically visible colonies were formed. Colonies were visualized by fixing cells in 4% PFA in PBS for 20 min, washed with PBS, and stained with 1% crystal violet in 20% ethanol for 20 min. Plates were washed with water and scanned. To test the migration potential, 100,000 HCT116 cells were seeded per PET track-etched membrane cell culture insert (8 μm, BD Bioscience) which was equilibrated with serum-free growth medium for 30 min. After 48 h, the inside of the inserts was scraped with a humidified cotton swab to remove non-migrated cells. Afterwards, migrated cells were fixed in 100% methanol for 10 min, stained with 0.1% crystal violet in 20% ethanol for 20 min, and rinsed with water. The stained area (%) covered by cells was quantified using FIJI [17].

### qRT-PCR and Western blotting

RNA was isolated using TRIzol (Invitrogen) and reverse transcribed using Moloney Murine Leukemia Virus reverse transcriptase (New England Biolabs) and random primers. Gene expression analysis was performed by quantitative real-time PCR using SYBR Green I (Roche Diagnostics). All expression values were normalized to 18S rRNA levels and corresponding primers are listed in Additional file 3: Table S2. For Western blot analyses, cells were lysed in RIPA buffer (1% NP-40, 0.5% sodium deoxycholate and 0.1% SDS dissolved in PBS) containing protease, phosphatase, and deubiquitinase inhibitors (1 ng/μL Aprotinin/Leupeptin, 10 mM β-glycerophosphate, 1 mM N-ethylmaleimide, 1 mM Pefabloc) and sonicated for 15 min. Proteins were denatured in 6× Laemmli buffer at 95 °C for 5 min, separated by SDS-PAGE, and blotted onto nitrocellulose membranes. For RNA synthesis as well as protein isolation, floating and adherent cells were collected. Primary antibodies used for Western blot analyses are listed in Additional file 3: Table S3.

### mRNA-seq data analysis

Gene expression studies as well as Gene Set Enrichment Analysis (GSEA; [18, 19]) following RNF40 depletion were performed as previously described [15] (ArrayExpress accession number: E-MTAB-7197).

### Chromatin immunoprecipitation (ChIP)

To assess gene occupancy by H2Bub1, H3K27ac, and H3K79me3, SW837 cells were fixed in 1% formaldehyde in PBS for 20 min and quenched with 125 mM glycine for 5 min. Cells were scraped and washed with Nuclear Preparation Buffer (5 mM EDTA, 150 mM NaCl, 0.5% (*v*/*v*) NP-40, 50 mM Tris/HCl (pH 7.5), 1% (*v*/*v*) Triton X-100). The nuclear pellet was lysed using Lysis Buffer (20 mM EDTA, 150 mM NaCl, 1% (*v*/*v*) NP-40, 0.5% SDS, 0.5% (*w*/*v*) sodium deoxycholate, 20 mM sodium fluoride, 50 mM Tris/HCl (pH 8.0) and protease inhibitors) and sonicated for 20 cycles (30 s on/off). Samples were precleared with 50% Sepharose 4B (GE Healthcare) and incubated with the respective antibodies overnight (H3K27ac (196-050, Diagenode), H3K79me3 (C15310068, Diagenode) and H2Bub1 (5546, Cell Signaling)). Sepharose beads with Protein A (GE Healthcare) were added and incubated for 2 h, and immunocomplexes were washed twice with Wash Buffer (20 mM EDTA, 500 mM LiCl, 1% (*v*/*v*) 20 mM NaF, NP-40, 1% (*w*/*v*) sodium deoxycholate, 100 mM Tris (pH 8.5)) and twice with TE buffer. Next, samples were incubated with RNase A (Qiagen) in 10 mM Tris (pH 8.0) at 37 °C for 30 min. For de-crosslinking, samples were incubated in 20 mM EDTA, 2% SDS, 100 mM Tris/HCl (pH 8), and 20 μg Proteinase K at 65 °C for 4 h. Samples were extracted with Roti® phenol/chloroform/isoamylalcohol (Roth) and DNA was precipitated with ethanol. The corresponding input samples were used as controls.

### ChIP-seq and data analysis

ChIP libraries were prepared using the NEBNext® Ultra DNA library preparation Kit (NEB) according to the manufacturer’s instructions and samples were sequenced (single-end 50 bp) on a HiSeq2000 (Illumina). Quality control of fastq files was performed via FastQC (version 0.72), and reads were mapped to the human reference genome (GRCh38.p10) using Bowtie2 (version 2.3.4.2) with very sensitive presets in end-to-end mode on Galaxy. Reads were normalized to reads per kilobase per million (RPKM) by the bamCoverage tool (version 3.2.0.0.0) ignoring duplicates and extending for 125 base pairs. Using bigwigCompare (version 3.2.0.0.0), the signal of the input controls was subtracted from the respective data sets. Occupancy profiles were viewed by the Integrative Genomics Viewer (IGV 2.5.0) [20]. All ChIP-seq data have been deposited at ArrayExpress (http://www.ebi.ac.uk/arrayexpress, accession number: E-MTAB-7786).

### Cell cycle analysis

To analyze the cell cycle profile, adherent cells were trypsinized, washed with PBS, and fixed with 70% ice-cold ethanol overnight. Cell pellets were resuspended in PBS containing 10–20 μg/ml RNase (Macherey-Nagel GmbH & Co. KG) and 20–40 μg/ml propidium iodide (Sigma Aldrich). Samples were incubated at room temperature in the dark for 30 min. Cell cycle profiles were obtained using the Guava flow cytometry system (Millipore) using the same gate settings for each cell line. Three biological replicates with each two technical replicates were processed in three independent experiments.

### Annexin V assay

Floating cells were collected and adherent cells were trypsinized, filtered, and washed with ice-cold PBS. After centrifugation, 100,000 cells were resuspended in 100 μl binding buffer (0.01 M HEPES pH 7.4, 0.14 M NaCl, 2.5 mM CaCl_2_). Propidium iodide (Sigma Aldrich) and Annexin V-FITC (Southern Biotech) were added (1:100) and incubated at room temperature for 15 min. Four hundred microliters of binding buffer was added, and apoptosis analyses were performed using the Guava flow cytometry system (Millipore) using the same gate settings for each cell line. The experiment was performed with three biological replicates per condition, each with two technical replicates in three independent experiments.

### ViaStain^TM^ apoptosis assay

To evaluate caspase 3/7 activity, a Celigo® S-based ViaStain^TM^ (Nexcelom Bioscience) apoptosis assay was carried out. An endpoint analysis was performed according to the manufacturer’s instructions 72 h after the siRNA-mediated knockdown of RNF40.

### Statistical analyses

All graphs in this study were made with GraphPad Prism version 5.04 (GraphPad Software, Inc.). Statistical analysis was performed using the one-way analysis of variants (ANOVA) and Tukey post hoc test (*α* = 0.05, *p ≤ 0.05, **p ≤ 0.01,***p ≤ 0.001).

## Results

### RNF40 knockdown reduces growth and tumorigenic features of CRC cell lines

**Fig. 1:**
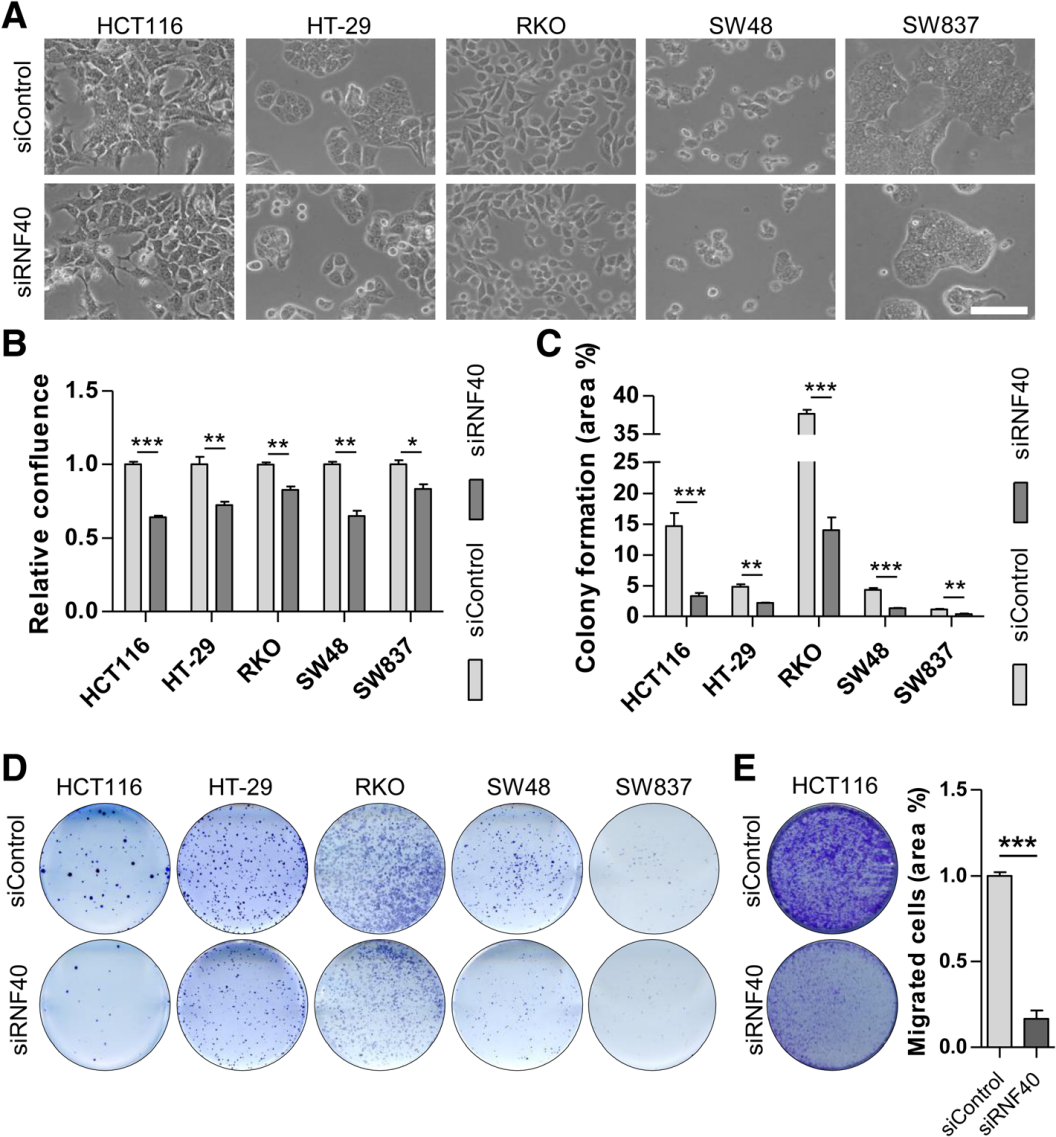
RNF40 loss reduces the tumorigenic properties of CRC cells in vitro. Functional assays were performed using HCT116, HT-29, RKO, SW48, and SW837 cells after siRNA-mediated RNF40 knockdown in three independent experiments. **a** The morphology of CRC cell lines was not affected 72 h after RNF40 knockdown. Scale bar, 1 mm. **b** Cellular confluence was measured 72 h post-transfection using a Celigo® S device and decreased cell numbers were detected following RNF40 depletion (*n* = 6). Mean ± SEM, Students *t* test. **c** The clonogenic capacity of CRC cells was determined by quantifying well areas covered by crystal violet-stained colonies formed from single cells (*n* = 3). Upon loss of RNF40, the cells formed fewer colonies. Mean ± SEM, Students *t* test. **d** Representative images of the colony formation assay. **e** Migration potential was significantly decreased in HCT116 cells with reduced RNF40 levels. Mean ± SEM, Students *t* test

Our previous studies suggested that RNF40 might be required for the pro-proliferative behavior of colorectal cancer cells in vitro [15]. To test the general impact of RNF40 reduction on CRC cells, we examined the morphology of HCT116 and four additional CRC cell lines not previously tested following siRNA-mediated knockdown of RNF40 (siRNF40) compared to control cells (siControl). The knockdown efficiency was defined at the mRNA and protein levels (Additional file 1: Figure S1A, B). As we previously observed in other CRC cell lines, while the gross morphology was not affected by RNF40 knockdown 72 h post-transfection (Fig. 1a), Celigo® S-based quantification revealed that the confluence was significantly reduced in siRNF40 cells (Fig. 1b). We next evaluated the effect of RNF40 knockdown on the clonogenic capacity of the investigated cell lines. Indeed, the ability of single cells to form colonies was significantly reduced following RNF40 knockdown (Fig. 1c, d). In addition, the migration potential of HCT116 RNF40 knockdown cells was significantly reduced compared to the control cells (Fig. 1e). In summary, these findings demonstrate that the loss of RNF40 leads to reduced cell confluence as well as decreased tumorigenic features of all CRC cell lines tested.

### Permanent CRISPR/Cas9-mediated RNF40 deletion is lethal in CRC cells in vitro

In order to validate our observations upon depletion of RNF40 by siRNA, we sought to establish a permanent RNF40 deletion in CRC cells using CRISPR/Cas9-mediated gene editing. For this purpose, we used an approach to delete both exons 3 and 4 by using two guide RNAs (gRNAs) targeting the intron regions between exon 2 and 3 and exons 4 and 5, respectively. This approach was chosen since it specifically mirrors the conditional *Rnf40* knockout mouse model previously described by targeting the homologous regions and exons of the human *RNF40* gene [15, 21]. Importantly, the resulting loss of exons 3 and 4 will lead to a frameshift of any resulting spliced transcript, thereby ensuring the lack of functionality of any resulting protein (Additional file 2: Figure S2A). To validate the effectiveness of *RNF40* deletion, we transfected SW480 cells with RNF40 gRNAs cloned in a GFP-fused Cas9 plasmid (pSpCas9(BB)-2A-GFP; PX458, Addgene) using Lipofectamine 2000® and examined RNF40 expression by immunofluorescent staining. Interestingly, 24 h after transfection, we observed that GFP-expressing cells were devoid of RNF40 expression, validating the efficiency of our approach (Additional file 2: Figure S2B). We subsequently utilized electroporation to obtain a higher efficiency of transfection, and after 48 h, we sorted single, highly positive GFP-expressing cells by flow cytometry into single wells in a 96-well plate format to obtain single cell clones. While we were able to readily sort cells based on high GFP expression (Additional file 2: Figure S2C), not a single resulting clone (out of 42) was positive for a homozygous deletion of *RNF40* (Additional file 1: Figure S1D). Alternative approaches utilizing gRNAs directly targeting different exons (exons 2, 3, 6, and 9) also failed to produce any homozygous clones (data not shown). This pattern of lethality was also observed in MDA-MB-231 breast cancer cells, which also failed to produce any knockout clones even after screening ca. 150 clones (Additional file 2: Figure S2D). Together, these data implied that the complete and permanent loss of *RNF40* is lethal in CRC cells in vitro. Therefore, in order to further investigate the molecular mechanisms underlying this lethality, all subsequent experiments were performed using a transient, siRNA-mediated knockdown of RNF40.

### RNF40 loss does not affect cell cycle progression in CRC cells

**Fig. 2:**
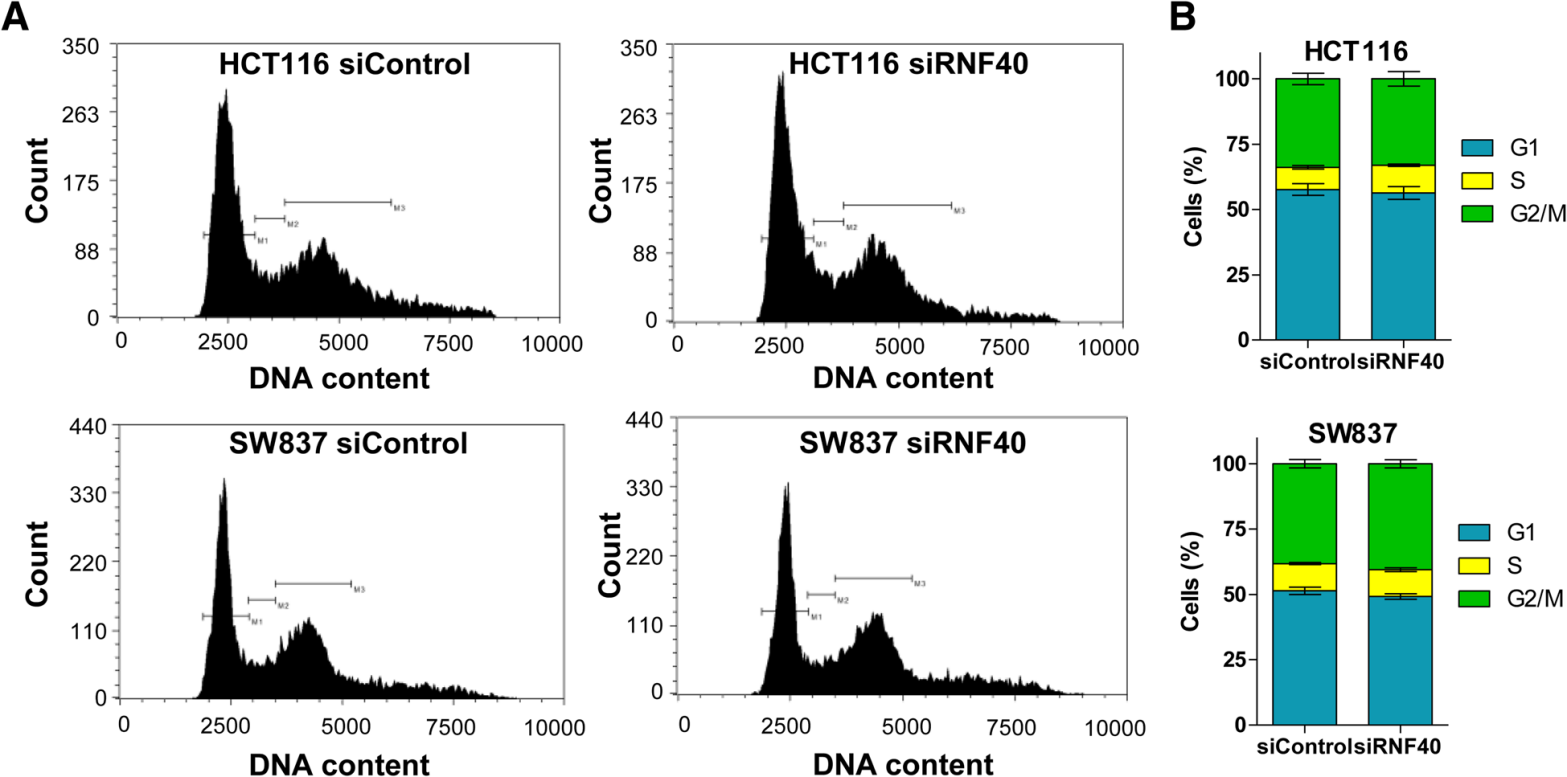
RNF40 depletion does not significantly affect the cell cycle profile of CRC cells. Adherent HCT116 and SW837 cells were harvested 72 h after siRNA-mediated knockdowns in three independent experiments (*n* = 3). **a** Representative images of flow cytometry profiles of the DNA content using propidium iodide staining. **b** The proportions of cells in the respective cell cycle phases were determined in HCT116 and SW837 cells and no significant differences were detected between RNF40 control and knockdown conditions. Mean ± SEM

To elucidate the mechanisms underlying the decreased cell number following RNF40 depletion (or deletion), we evaluated gene expression signatures enriched following RNF40 depletion by performing Gene Set Enrichment Analysis (GSEA) of our previously published mRNA-seq dataset in HCT116 cells [15]. Previous analyses uncovered a regulation of cell cycle-associated gene signatures related to cell division, cell cycle phase transition, and DNA replication as being negatively enriched in HCT116 RNF40 knockdown cells. Consequently, we sought to evaluate the effects of RNF40 knockdown and, consequently, the reduction on H2Bub1 levels (Additional file 1: Figure S1C) on the cell cycle. For this purpose, we tested the proportions of adherent HCT116 and SW837 control and RNF40 knockdown cells in the respective phases of the cell cycle using propidium iodide staining. Surprisingly, we did not detect any significant changes in the flow cytometry profiles as well as in proportions of cells in the respective cell cycle phases after RNF40 knockdown in either cell line (Fig. 2a, b). Thus, despite the regulation of cell cycle-associated genes, the distribution of cells in the respective cell cycle phases was not appreciably affected by RNF40 depletion.

### RNF40 knockdown cells display apoptotic features

**Fig. 3:**
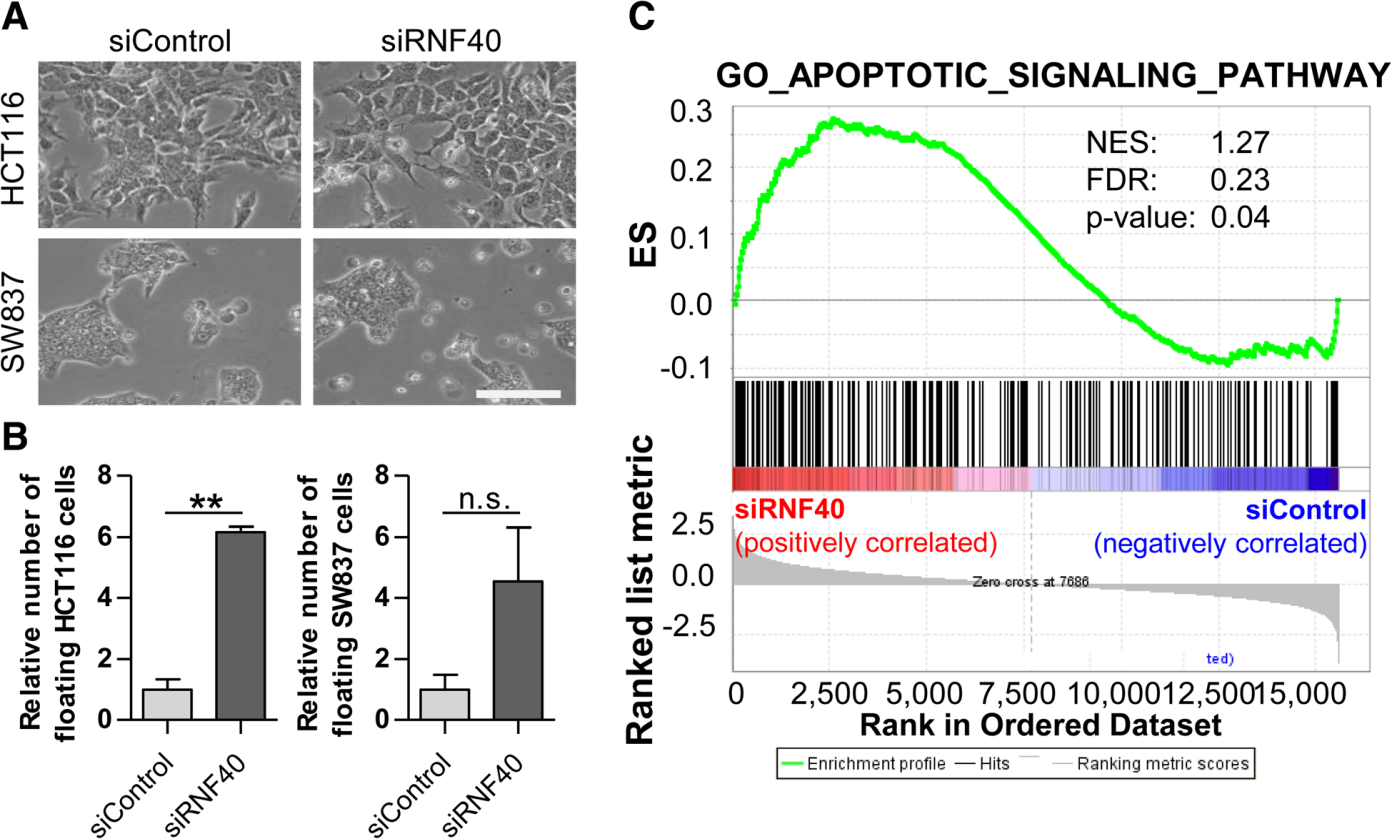
Cell morphology and differentially regulated genes suggest apoptotic processes in RNF40 knockdown cells. **a** A higher proportion of non-adherent cells was observed after the siRNA-mediated knockdown of RNF40. Scale bar, 1 mm. **b** The same number of cells was seeded prior to the knockdown, and 72 h later, the number of floating and attached cells was counted in three independent experiments. The relative number of non-adherent cells was significantly higher after the loss of RNF40. Mean ± SEM, Student’s *t* test. **c** Gene set enrichment analysis indicated increased apoptotic processes after RNF40 knockdown in HCT116 cells

Since only negligible effects of RNF40 knockdown on the cell cycle could be observed, we aimed to elucidate further processes underlying the decreased confluence of siRNF40 cells. We observed high numbers of non-adherent cells in the medium upon RNF40 loss (Fig. 3a). Thus, after seeding the same number of cells for control and knockdown cells, we quantified the respective cell populations. Indeed, the relative number of floating cells was four to sixfold increased 72 h after RNF40 depletion (Fig. 3b). Moreover, in our HCT116 GSEA data, we observed an enrichment of apoptosis-associated genes after the loss of RNF40 (Fig. 3c).

### RNF40 depletion promotes apoptosis

**Fig. 4:**
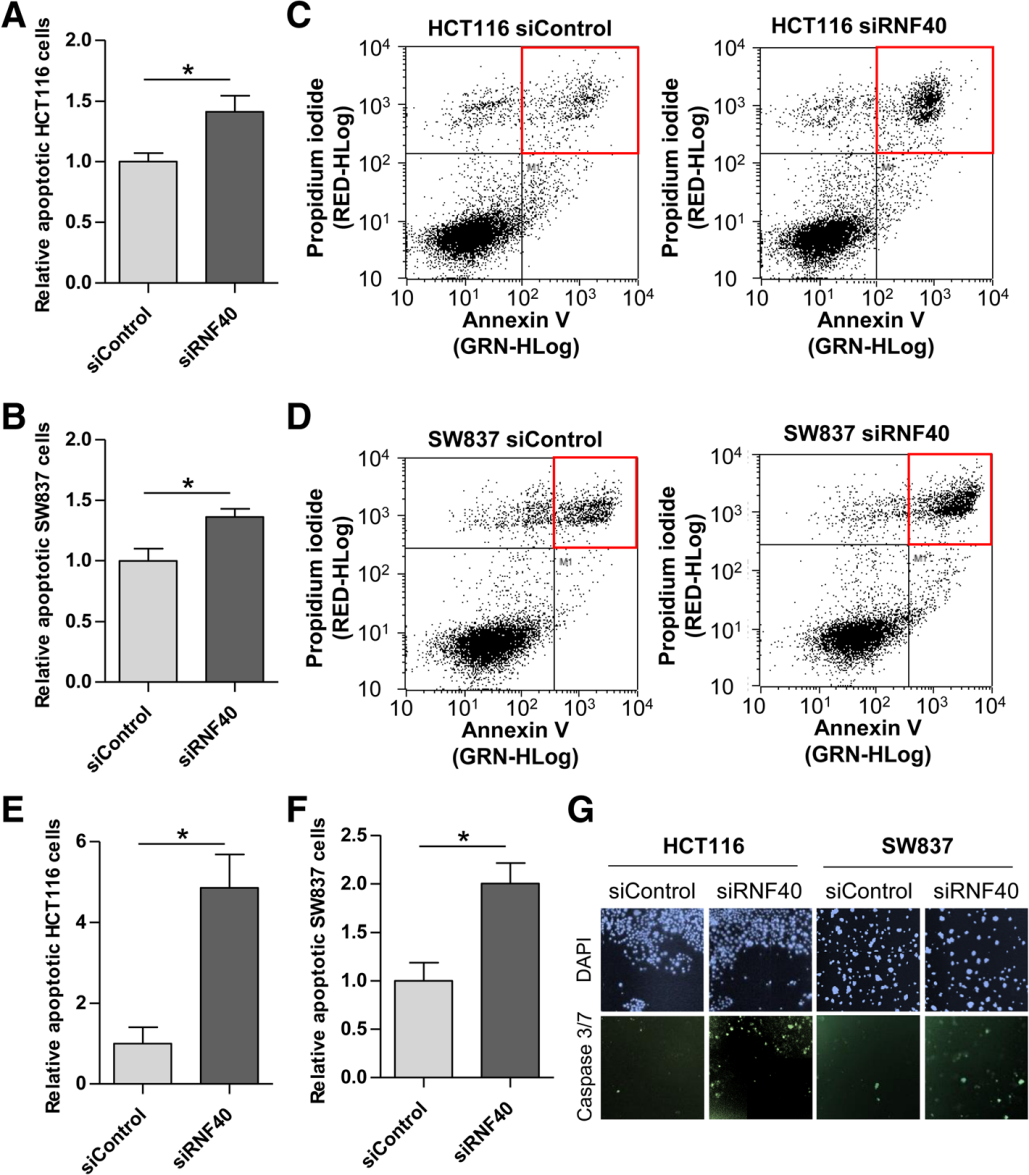
The loss of RNF40 promotes apoptosis in CRC cells. **a** HCT116 and **b** SW837 cells were stained with Annexin V-FITC and propidium iodide and analyzed using the Guava flow cytometry system (Millipore) 72 h after control or RNF40 knockdown. Three independent experiments were performed with three biological replicates per condition each with two technical replicates. The relative number of apoptotic cells was significantly increased in both cell lines after RNF40 knockdown. Mean ± SEM, Student’s *t* test. **c** Gated cells of the flow cytometry-based Annexin V assay in HCT116 and **d** SW837 cells. Red frames indicate gated late apoptotic cells. **e** Caspase 3/7 activity was measured using a Celigo® S-based ViaStain^TM^ (Nexcelom Bioscience) apoptosis assay and confirmed increased apoptosis after the loss of RNF40 in HCT116 and **f** SW837 cells. Mean ± SEM, Student’s *t* test. **g** Representative images of the ViaStain^TM^ assay indicating DAPI-stained nuclei (blue) and cells with caspase 3/7 activity (green)

Next, we aimed to investigate in more detail whether reduced RNF40 levels promote apoptosis in CRC cells in vitro. SW837 and HCT116 siControl and siRNF40 cells were stained with Annexin V-FITC and propidium iodide 72 h after siRNA transfection to measure the proportion of late apoptotic cells. Indeed, we found an increase in the fraction of apoptotic cells following RNF40 depletion in both cell lines (Fig. 4a–d). To verify these findings, we performed a Celigo® S-based ViaStain^TM^ (Nexcelom Bioscience) apoptosis assay. Here, cells were incubated with a specific probe (coupled to a fluorescent dye) which is capable of binding to DNA. Upon caspase 3/7-mediated apoptosis, the complex is cleaved, thereby releasing the dye which translocates to the nucleus where it generates a fluorescent signal. In support of our Annexin V staining, we were able to detect an increase in apoptotic cells following RNF40 knockdown using this approach (Fig. 4e–g). While only a slight but significant increase in caspase 3/7 activity was detected in SW837 cells, the increase of apoptotic cells in HCT116 cells was nearly fivefold. Together, our data clearly demonstrate that reduced expression of RNF40 promotes apoptosis of CRC cells.

### RNF40 knockdown increases apoptosis by affecting BCL-2 family members

**Fig. 5:**
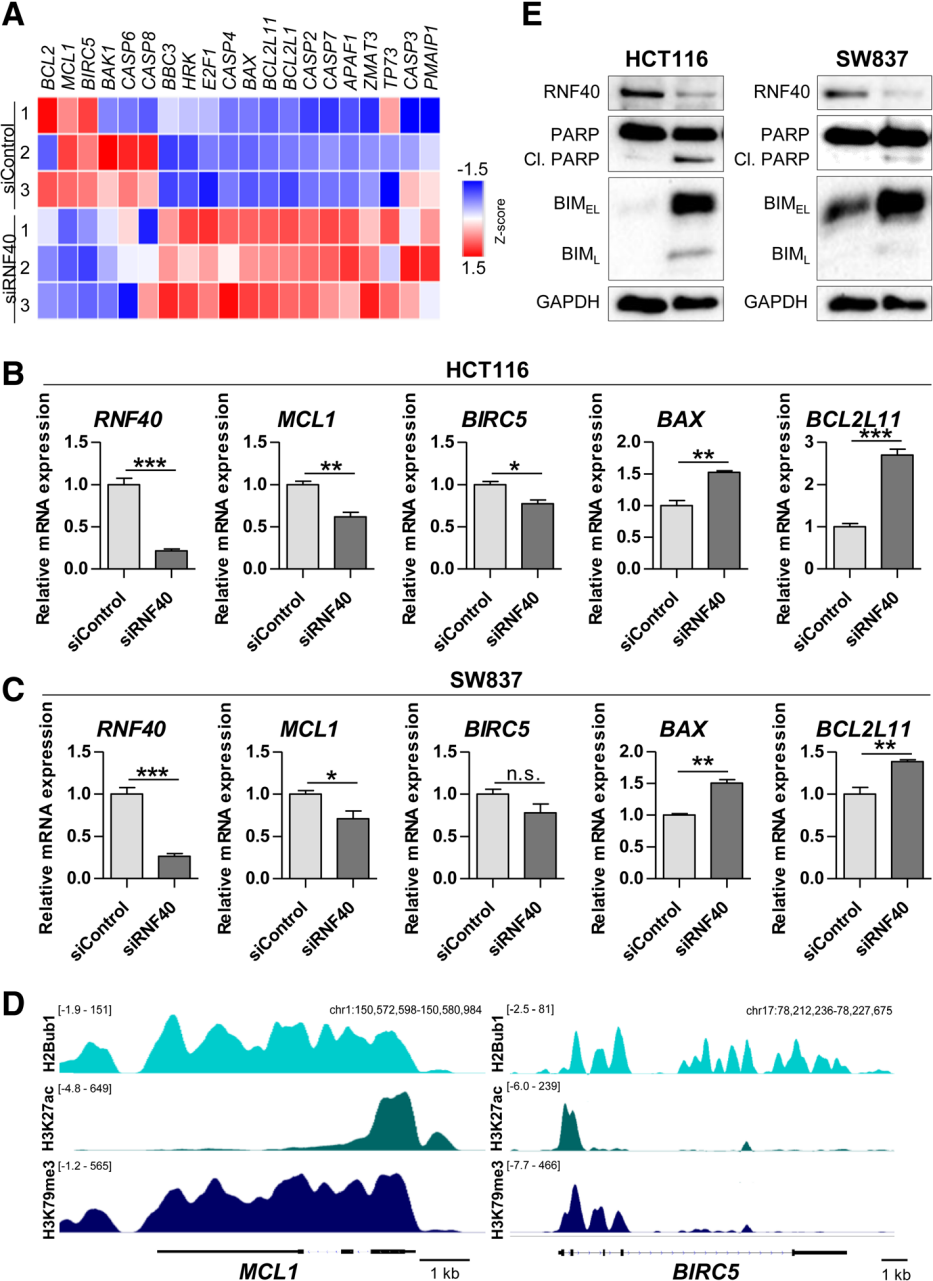
RNF40 knockdown promotes apoptosis by affecting BCL-2 family members. **a** Heatmap indicating gene expression levels based on mRNA-seq after siRNA-mediated knockdown of RNF40 in HCT116 cells. RNF40 loss resulted in a deregulated expression of several BCL-2 family members. **b** Verification of mRNA-seq results in HCT116 cells using qRT-PCR in three independent experiments. RNF40-depleted HCT116 cells display decreased mRNA levels of the anti-apoptotic factors *MCL1* and *BIRC5* while pro-apoptotic genes *BAX* and *BCL2L11* were upregulated (*n* = 3); **c** these findings were confirmed in SW837 cells. Mean ± SEM, Student’s *t* test. **d** ChIP-seq profiles show the occupancy of H2Bub1, H3K27ac, and H3K79me3 on the anti-apoptotic genes *MCL1* and *BIRC5* in SW837 cells. Input signals were subtracted. **e** In three independent experiments, protein was harvested from HCT116 and SW837 cells and analyzed using Western blot. In both cell lines, RNF40 loss elevated the protein amounts of BIM (encoded by *BCL2L11*) and the apoptosis marker cleaved PARP

To gain mechanistic insights into how the siRNA-mediated depletion of RNF40 promotes apoptosis, we analyzed our HCT116 mRNA-seq data for the expression of several pro- and anti-apoptotic genes. Interestingly, several members of the BCL-2 family were differentially regulated at the mRNA level in RNF40-depleted HCT116 cells (Fig. 5a). While anti-apoptotic factors such as *BCL2*, *MCL1*, and *BIRC5* were downregulated upon the loss of RNF40, pro-apoptotic genes (e.g., *BAX*, *BCL2L11*, and *HRK*) were upregulated. These findings could be confirmed by qRT-PCR which confirmed that *BCL2L11* mRNA levels were increased up to threefold in RNF40 knockdown cells (Fig. 5b, c). As described earlier by our group, H2Bub1 occupancy is highly associated with gene expression and active histone marks such as H3K27ac and is required for downstream methylation of lysine 79 of histone H3[21]. Consistent with the positive role of RNF40-mediated H2B monoubiquitination in controlling gene expression, ChIP-seq analysis in SW837 cells revealed high occupancy of H2Bub1 and H3K79me3, as well as H3K27ac, on the RNF40-dependent anti-apoptotic genes *MCL1* and *BIRC5* (Fig. 5d). Moreover, as detected by Western blot analysis, BIM (encoded by *BCL2L11*) was substantially increased in siRNF40 cells, a finding which was confirmed in SW837 cells. Moreover, we assessed the levels of poly (ADP-ribose) polymerase (PARP) and detected increased amounts of cleaved PARP (Fig. 5e). Generally, PARP is a substrate of active caspases and, therefore, high cleaved PARP levels confirmed our ViaStain^TM^ results that caspase-mediated apoptotic processes are increased in RNF40-depleted cells. In summary, our findings suggest that RNF40 suppresses apoptosis by maintaining the expression of anti-apoptotic BCL-2 family members via H2B monoubiquitination on the transcribed region of these genes.

### Inhibition of caspase activity maintains viability of RNF40 knockdown cells

**Fig. 6:**
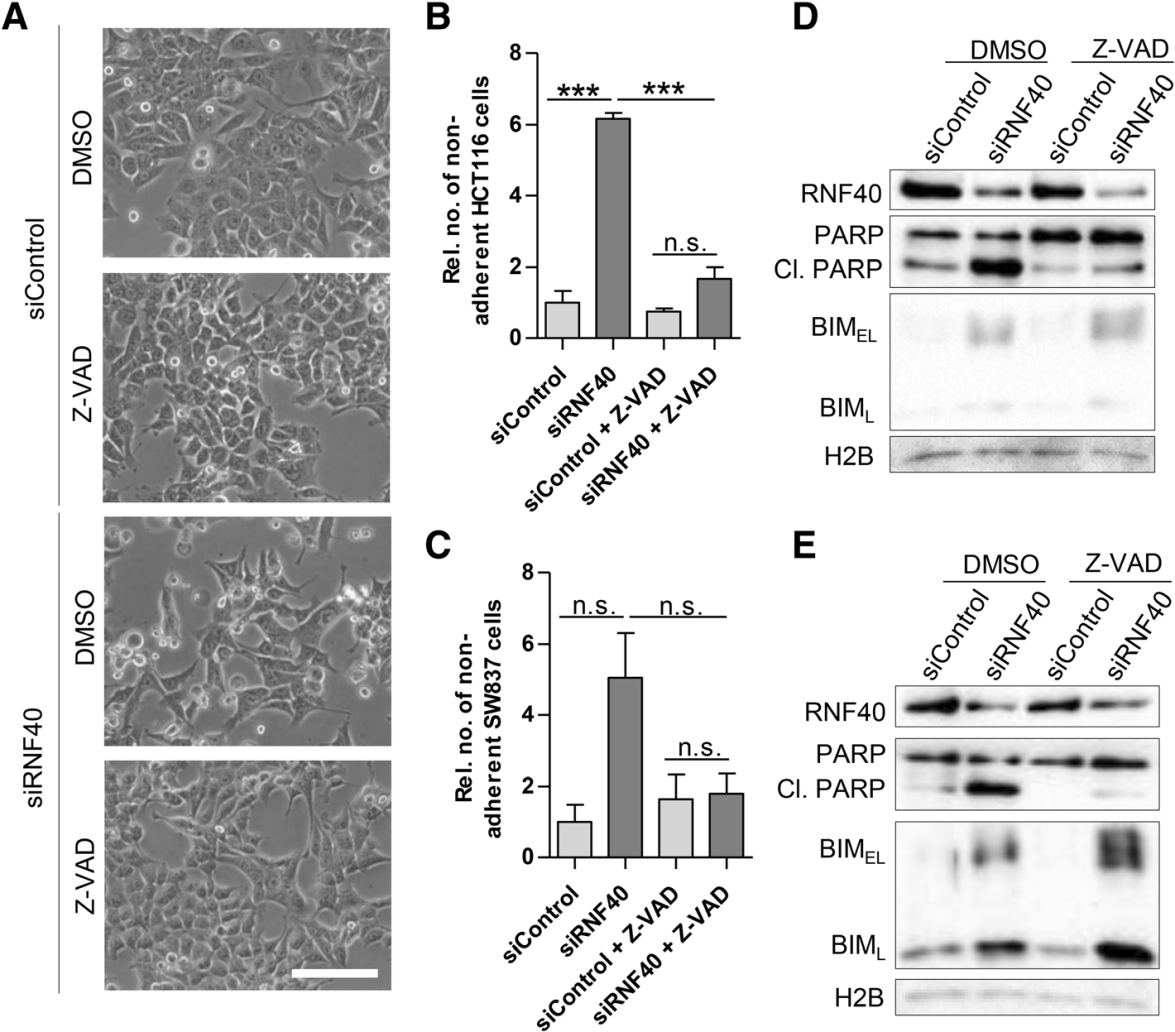
Caspase inhibition rescues apoptosis in RNF40 knockdown cells. Twenty-four hours after knockdown, HCT116 and SW837 cells were treated with 80 μM Z-VAD for 48 h to inhibit caspases in three independent experiments. **a** Morphologically, the appearance of siRNF40 cells was highly similar to the controls after Z-VAD treatment as exemplarily shown for HCT116 cells. Scale bar, 1 mm. **b** The percentage of adherent and detached cells was assessed. The proportion of dead cells was significantly reduced in HCT116 cells and a similar trend was observed in **c** SW837 RNF40 knockdown cells. Mean ± SEM, one-way ANOVA. **c** Western blot analysis revealed that Z-VAD treatment decreased the levels of cleaved PARP, while BIM remained unchanged after RNF40 knockdown in HCT116 and **e** SW837 cells

Based on the elevated caspase 3/7 activity and the increase in cleaved PARP upon RNF40 knockdown, we hypothesized that the apoptotic phenotype of these cells can be rescued by inhibiting caspases. For this purpose, 24 h after siRNA transfection, HCT116 and SW837 cells were treated with 80 μM Z-VAD-FMK (herein referred to as Z-VAD) for 48 h. As expected, in DMSO-treated control cells, the loss of RNF40 resulted in lower cell numbers and the presence of a high proportion of detached cells (Fig. 6a). In contrast, after Z-VAD treatment, less floating cells were visible. To quantitate the proportions of adherent and non-adherent cells, we counted all cells floating in the medium (Fig. 6b, c). While Z-VAD treatment showed only minimal effects on siControl cells, the number of viable cells was significantly elevated by Z-VAD in RNF40-depleted cells. These findings were confirmed by Western blot analysis where PARP cleavage could be substantially reduced, while BIM levels remained unchanged (Fig. 6d, e). Together, these results support the findings that RNF40 loss enhances apoptosis in CRC cells due to increased caspase activity.

### RNF40 loss reduces tumorigenic properties independent of apoptosis regulation

**Fig. 7:**
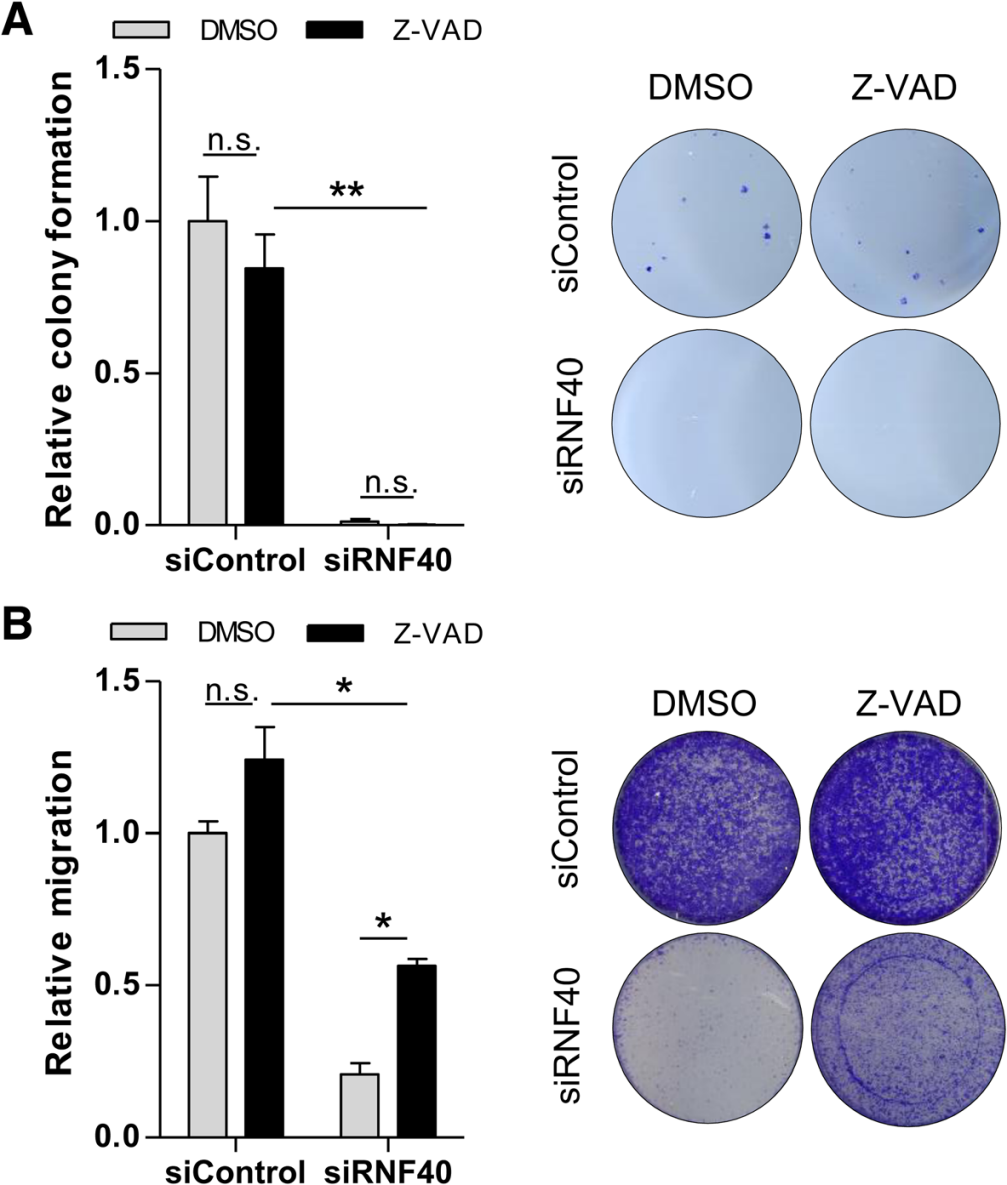
RNF40 knockdown cells with restored growth capacity show decreased tumorigenic properties. The effect of RNF40 knockdown in combination with Z-VAD treatment was tested in three independent experiments. **a** The clonogenic potential of HCT116 siRNF40 cells was reduced compared to wild-type controls. This phenotype was not rescued after Z-VAD-mediated caspase inhibition (*n* = 2). Mean ± SEM, one-way ANOVA. **b** Migration potential was tested using a trans-well migration assay for 48 h. Z-VAD treatment increased the proportion of migrating cells under RNF40 knockdown conditions which was still significantly lower compared to wild-type cells (*n* = 3). Mean ± SEM, one-way ANOVA

After determining that the apoptotic phenotype of RNF40-depleted cells could be rescued by caspase inhibition, we aimed to determine whether RNF40 may have independent roles in controlling apoptosis and other tumorigenic features. Thus, we performed additional functional assays in HCT116 cells in the presence of Z-VAD to inhibit caspase activity and subsequent cell death. First, we carried out colony formation assays to test the capacity of single cells to form colonies. As expected, siRNF40 cells formed fewer colonies than the controls; however, this phenotype was not rescued after Z-VAD treatment (Fig. 7a). Next, we evaluated the migration capacity, which was significantly reduced after the loss of RNF40 (Fig. 7b). After restoring cell growth using Z-VAD, we were indeed able to slightly increase the number of migrated cells following RNF40 knockdown which was, however, still significantly lower compared to wild-type controls. In summary, even after rescuing the increased apoptosis in RNF40 knockdown cells, RNF40 depletion still reduces additional tumorigenic properties of CRC cells in vitro.

## Discussion

A tight regulation of apoptotic signaling is central to maintaining tissue homeostasis and a loss of the balance between cell death and survival and evasion of apoptosis is a common feature of cancer cells [4]. In this study, we were able to demonstrate that the knockdown of the E3 ligase RNF40 and, consequently, the loss of H2Bub1 results in reduced tumorigenic potential and increased apoptosis in human colorectal cancer cells in vitro.

H2Bub1 loss was also connected to cell death in previous studies. For instance, knockdown of a RNF20/RNF40 homolog in *Caenorhabditis elegans* resulted in germ cell apoptosis [22]. In addition, Duan and colleagues investigated the relevance of these E3 ligases in mitosis and spindle assembly [23]. They discovered that a loss-of-function of RNF20/RNF40 resulted in G2/M arrest and apoptosis by downregulating the motor protein Eg5 in the human breast cancer cell line MCF-7. Moreover, in TNFα-treated mammary epithelial cells, the knockdown of RNF20 reduced the expression of the anti-apoptotic factor *BCL-2* in vitro [11]. All these results suggest an anti-apoptotic function of RNF40. While we did not observe any cell cycle changes in SW837 and HCT116 cells, we were able to confirm that loss of RNF40 induces apoptosis.

Using qRT-PCR and ChIP-seq, we demonstrated that the loss of RNF40, presumably via decreased H2Bub1 gene occupancy, modulates the expression levels of various BCL-2 family members, thereby promoting apoptosis. Interestingly, several reports described the interplay between BCL-2 members and nuclear factor kappa-light-chain-enhancer of activated B cells (NF-κB), and it was suggested that NF-κB activity enhances the expression of anti-apoptotic genes [24–26]. This postulation is in agreement with our previous findings that the knockdown of RNF40 delayed the nuclear translocation of the NF-κB member RelA after TNFα treatment [15]. Consequently, the expression of NF-κB target genes was decreased after the loss of either RNF40 or RNF20. Future studies will provide further insight into the interplay between RNF40, BCL-2 family members, and NF-κB.

Besides the role of RNF40 in apoptosis, we investigated the effects of RNF40 depletion on other tumorigenic features of CRC cells in vitro. Our earlier findings implicated that RNF40 exerts oncogenic functions and promotes aggressive growth of CRC cells in vitro [15]. Here, we were able to verify in additional CRC cell lines that RNF40 knockdown reduces proliferation as well as clonogenic and migration potential. To rule out the possibility that increased apoptosis rates underlie the reduced cell growth of RNF40-depleted cells, we rescued apoptosis-associated effects by inhibiting caspases using Z-VAD. Notably, even after rescuing RNF40-depleted cells from apoptosis by Z-VAD treatment, cells displayed decreased clonogenic and migratory potential. Therefore, we were able to support the hypothesis that RNF40 exerts oncogenic functions in CRC. This finding is not in agreement with other correlative studies which described that the loss of H2Bub1 in colorectal tumors was linked to advanced tumor grades and poor survival [14]. However, our own data demonstrated that colorectal tumors display a high degree of heterogeneity in H2Bub1 levels, while RNF40 amounts were constant in cancer lesions, thereby suggesting that RNF40 may play additional roles in CRC apart from the monoubiquitination of H2B. However, the underlying mechanisms of such an uncoupling remain to be elucidated but may be related to upstream signaling and/or downstream deubiquitination of H2Bub1.

## Conclusion

Together, our findings suggest that RNF40 exerts pro-tumorigenic functions in colorectal cancer in vitro by increasing clonogenic potential as well as by suppressing apoptosis. This occurs by the regulation of BCL-2 family members, which can either suppress or promote apoptosis. Further experimental procedures will help to elucidate further underlying molecular mechanisms employed by RNF40 to reduce apoptotic signaling and to determine whether RNF40 may serve as a therapeutic target for inducing apoptosis in colorectal cancer cells.

## Additional files

Additional file 1: **Figure S1.** Reduction of RNF40 and H2Bub1 in siRNF40 CRC cell lines. (A-B). The siRNA-mediated knockdown of RNF40 was verified at the mRNA level using qRT-PCR (A) and on protein level using western blot (B) in three independent experiments 72 h after transfection. Mean ± SEM, Students *t* test. (C) The knockdown of RNF40 resulted in decreased H2Bub1 levels 72 h after siRNA transfection. (ZIP 7432 kb)

Additional file 2: **Figure S2.** *RNF40* knockout is lethal in colorectal cancer cells in vitro. (A) Diagram showing the approach to establish a permanent RNF40 knockout SW480 cells. Two gRNAs were targeted at intron regions before exon 3 and after exon 4 leading to a frameshift and a non-functional protein product. (B) Immunofluorescence assay showing the lack of RNF40 detection (red) when expressing GFP (green) which indicates successful transfection with the Cas9 construct. Scale bar: 10 μM. (C) A scatter plot showing the GFP-positive cells in non-transfected cells and transfected cells. Single cells were picked from the P4 population which is the shows highest GFP expression. (D) PCR amplification product detection on an agarose gel with no clone showing only the expected band (312 bp) upon successful deletion of *RNF40*. Control: Non transfected triplicate samples (2wA-C) of cells transfected with empty vectors, 24 h A, B, C: triplicates of SW480 cells transiently transfected with Cas9 after 24 h, 120 h A,B,C: triplicates of SW480 cells transiently transfected with Cas9 after 120 h, clones starting with M are MDA-MB-231 RNF40 knockout clones shown as comparison, clone numbers for SW480 (letter of plate, well number). (ZIP 5542 kb)

Additional file 3: **Tables S1–S3,** Supplemental tables. (DOCX 16 kb)

## Data Availability

All mRNA-seq data which have been previously published [15] and ChIP-seq data are available at ArrayExpress (http://www.ebi.ac.uk/arrayexpress, accession numbers: E-MTAB-7197 and E-MTAB-7786).

## Abbreviations

BAX: BCL2-associated X
BCL2: B-cell CLL/lymphoma 2
BCL2L11: BCL2-like 11
BIM: Bcl-2-interacting mediator of cell death (encoded by BCL2L11)
BIRC5: Baculoviral IAP repeat-containing 5
ChIP-seq: Chromatin immunoprecipitation DNA sequencing
CRC: Colorectal cancer
DAPI: 4′,6-Diamidino-2-phenylindole
DMSO: Dimethyl sulfoxide
FACT: Facilitates chromatin transcription
GFP: Green fluorescent protein
gRNA: Guide RNA
GSEA: Gene Set Enrichment Analysis
H2Bub1: Histone H2B monoubiquitination at lysine 120
H3K27ac: Histone H3 acetylation at lysine 27
H3K79me3: Histone H3 trimethylation at lysine 79
HRK: Harakiri
HSC70: Heat shock protein 70
MCL1: Myeloid cell leukemia 1
mRNA-seq: mRNA-sequencing
NF-κB: Nuclear Factor kappa-light-chain-enhancer of activated B cells
PARP: Poly (ADP-ribose) polymerase
PBS: Phosphate-buffered saline
PFA: Paraformaldehyde
RNF20: RING finger protein 20
RNF40: RING finger protein 40
WAC: WW domain-containing adapter protein with coiled-coil
