# Supplementary figures and images for: The E3 ubiquitin ligase RNF40 suppresses apoptosis in colorectal cancer cells
Source: Clin Epigenetics. 2019 Jul 2;11:98. doi: 10.1186/s13148-019-0698-x (PMC6604314; doi:10.1186/s13148-019-0698-x)

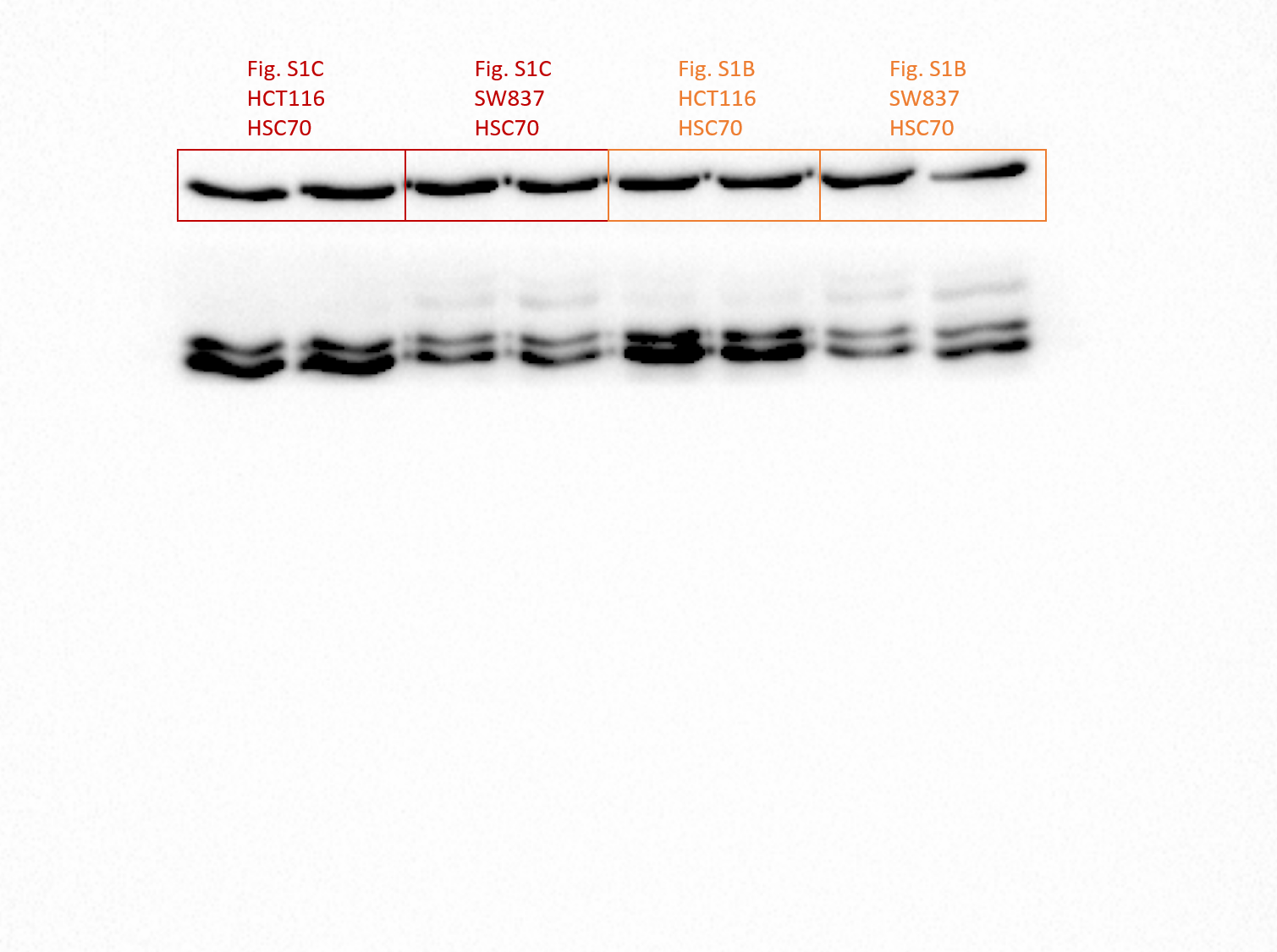

Supplement: Supplementary file 1 — Figure S1. Reduction of RNF40 and H2Bub1 in siRNF40 CRC cell lines. (A-B). The siRNA-mediated knockdown of RNF40 was verified at the mRNA level using qRT-PCR (A) and on protein level using western blot (B) in three independent experiments 72 h after transfection. Mean ± SEM, Students t test. (C) The knockdown of RNF40 resulted in decreased H2Bub1 levels 72 h after siRNA transfection. (ZIP 7432 kb) [file 13148_2019_698_MOESM1_ESM.zip › Fig.S1B,C_HCT116, SW837_HSC70_annotations.tif]

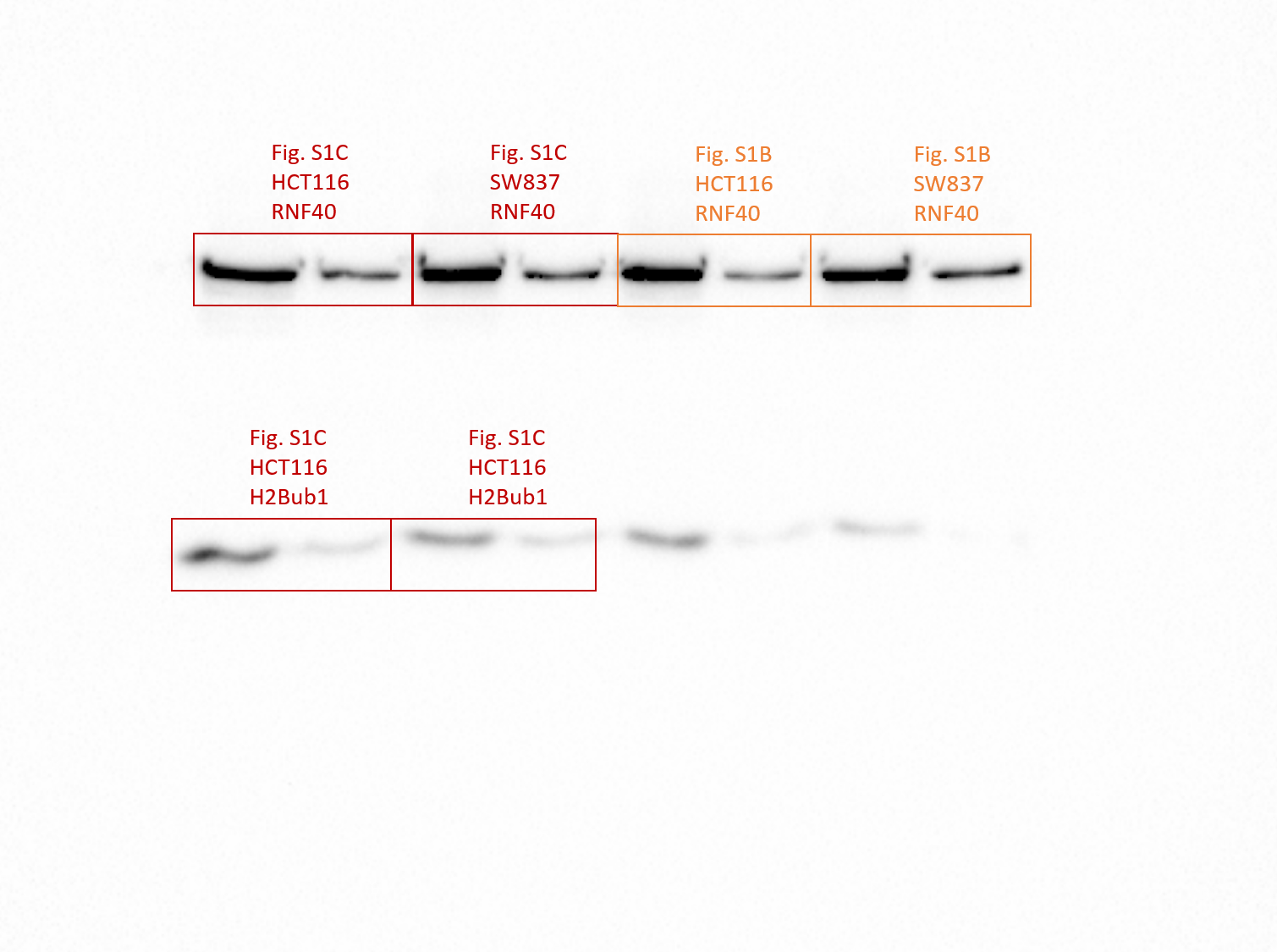

Supplement: Supplementary file 1 — Figure S1. Reduction of RNF40 and H2Bub1 in siRNF40 CRC cell lines. (A-B). The siRNA-mediated knockdown of RNF40 was verified at the mRNA level using qRT-PCR (A) and on protein level using western blot (B) in three independent experiments 72 h after transfection. Mean ± SEM, Students t test. (C) The knockdown of RNF40 resulted in decreased H2Bub1 levels 72 h after siRNA transfection. (ZIP 7432 kb) [file 13148_2019_698_MOESM1_ESM.zip › Fig.S1B,C_HCT116, SW837_RNF40, H2Bub1_annotations.tif]

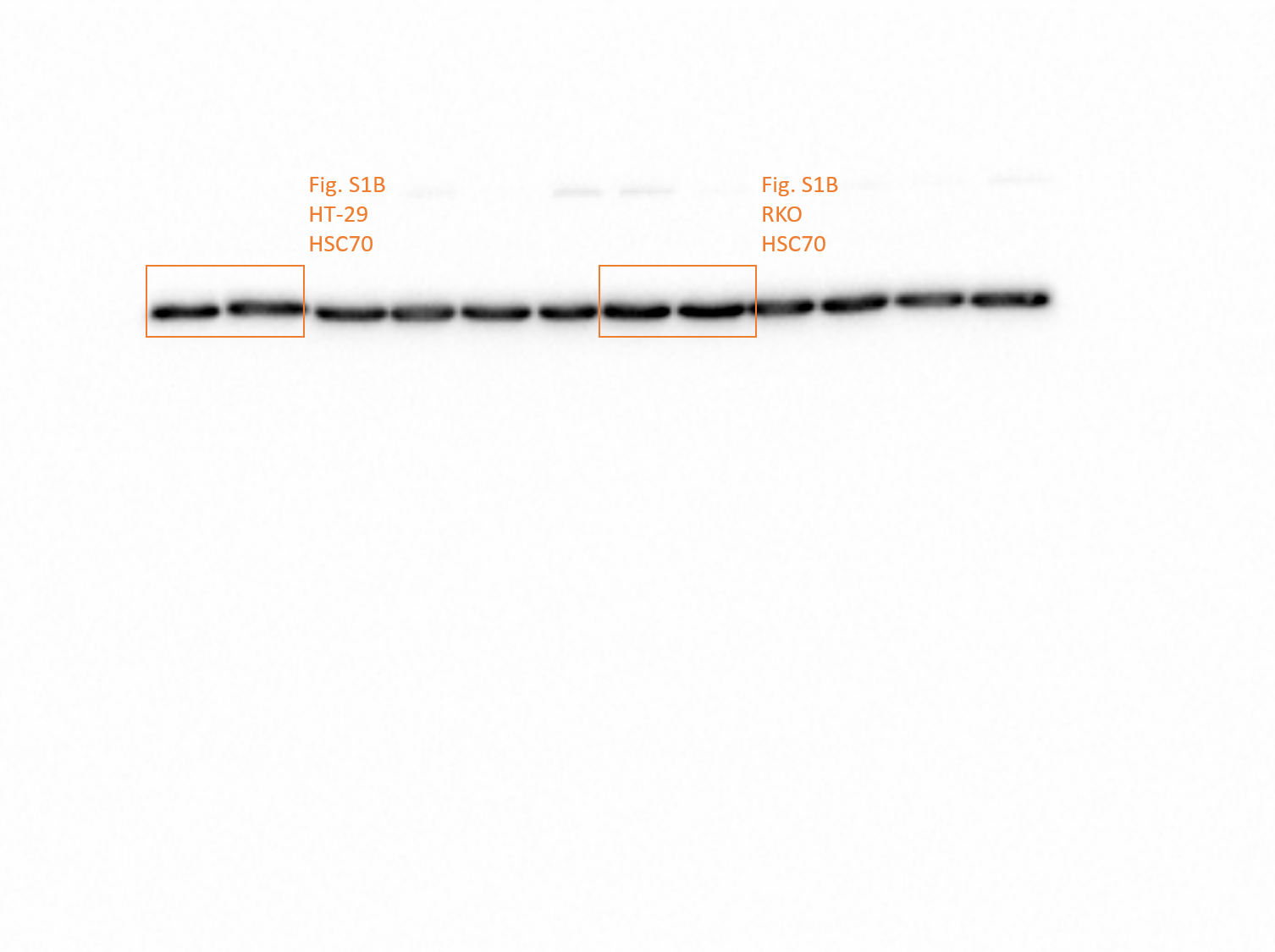

Supplement: Supplementary file 1 — Figure S1. Reduction of RNF40 and H2Bub1 in siRNF40 CRC cell lines. (A-B). The siRNA-mediated knockdown of RNF40 was verified at the mRNA level using qRT-PCR (A) and on protein level using western blot (B) in three independent experiments 72 h after transfection. Mean ± SEM, Students t test. (C) The knockdown of RNF40 resulted in decreased H2Bub1 levels 72 h after siRNA transfection. (ZIP 7432 kb) [file 13148_2019_698_MOESM1_ESM.zip › Fig.S1B_HT-29, RKO_HSC70_annotations.tif]

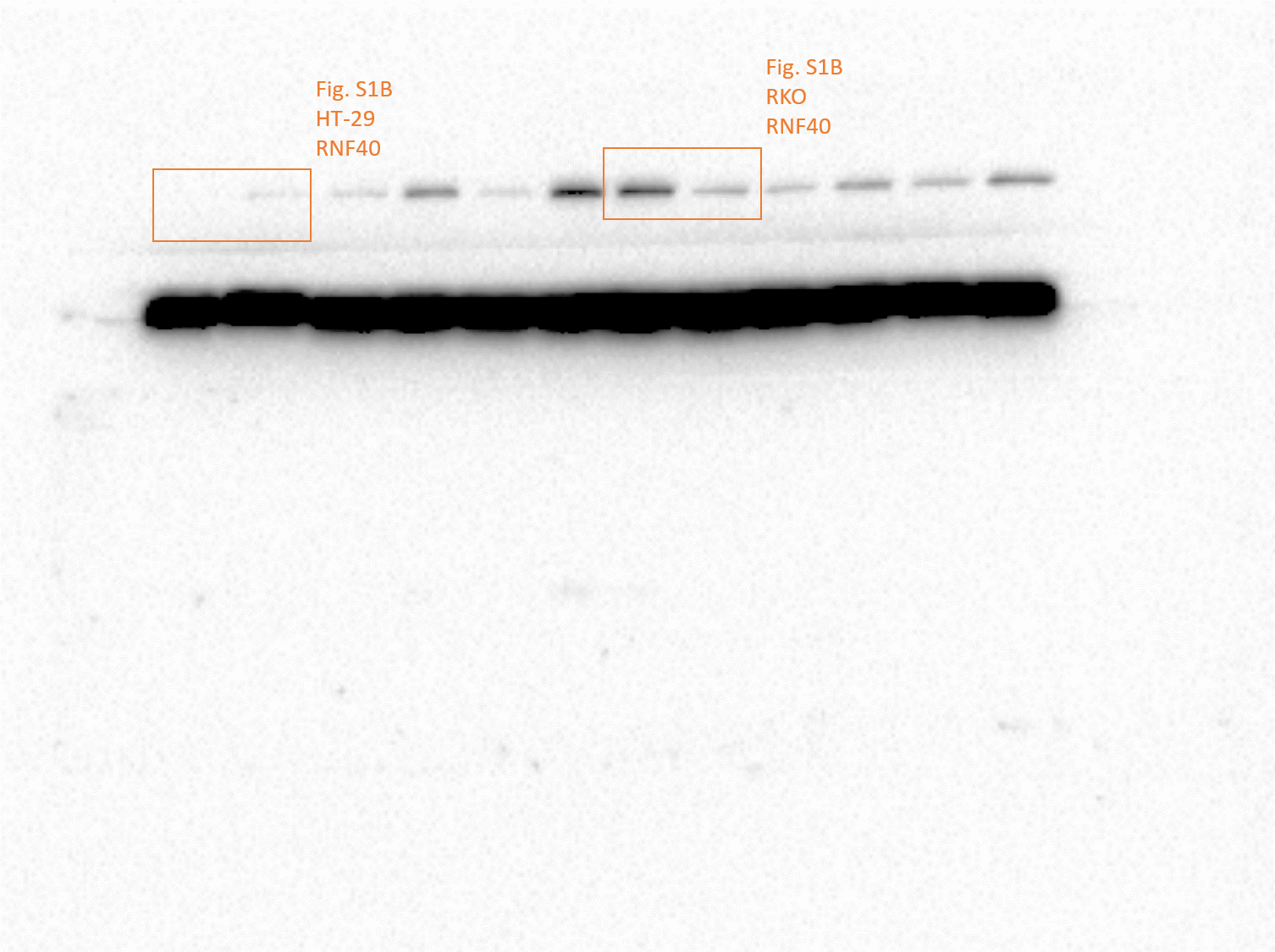

Supplement: Supplementary file 1 — Figure S1. Reduction of RNF40 and H2Bub1 in siRNF40 CRC cell lines. (A-B). The siRNA-mediated knockdown of RNF40 was verified at the mRNA level using qRT-PCR (A) and on protein level using western blot (B) in three independent experiments 72 h after transfection. Mean ± SEM, Students t test. (C) The knockdown of RNF40 resulted in decreased H2Bub1 levels 72 h after siRNA transfection. (ZIP 7432 kb) [file 13148_2019_698_MOESM1_ESM.zip › Fig.S1B_HT-29, RKO_RNF40_annotations.tif]

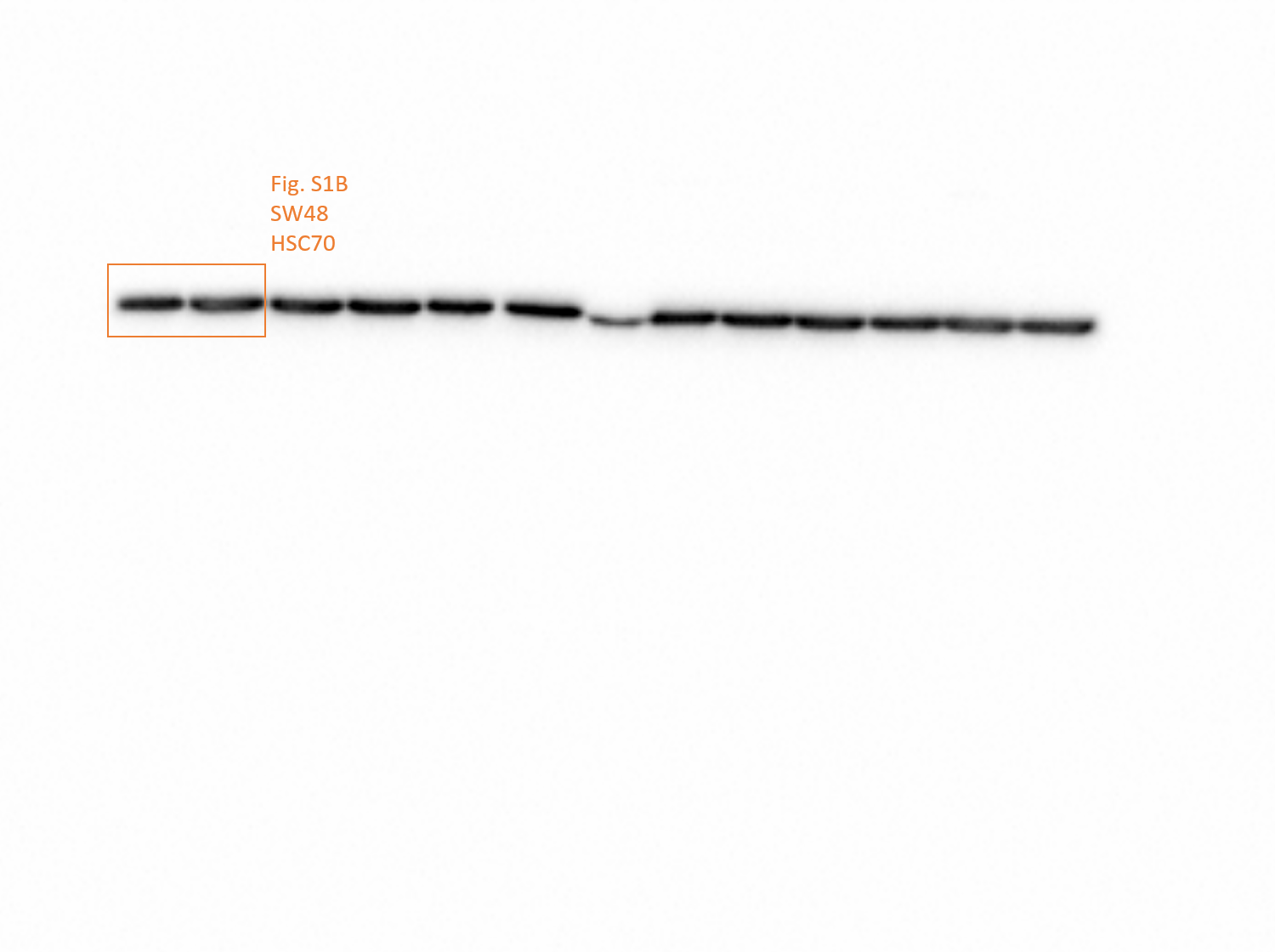

Supplement: Supplementary file 1 — Figure S1. Reduction of RNF40 and H2Bub1 in siRNF40 CRC cell lines. (A-B). The siRNA-mediated knockdown of RNF40 was verified at the mRNA level using qRT-PCR (A) and on protein level using western blot (B) in three independent experiments 72 h after transfection. Mean ± SEM, Students t test. (C) The knockdown of RNF40 resulted in decreased H2Bub1 levels 72 h after siRNA transfection. (ZIP 7432 kb) [file 13148_2019_698_MOESM1_ESM.zip › Fig.S1B_SW48_HSC70_annotations.tif]

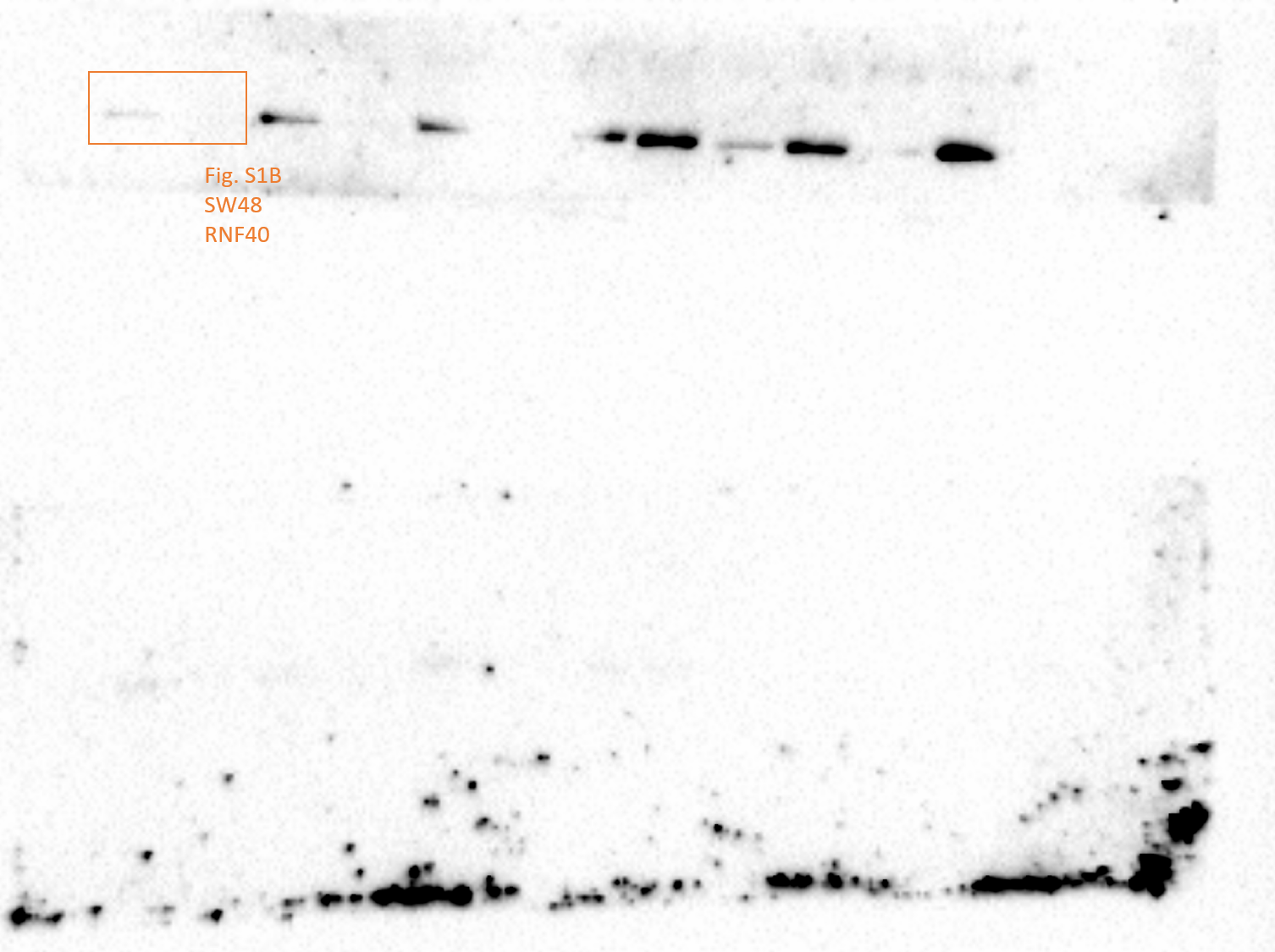

Supplement: Supplementary file 1 — Figure S1. Reduction of RNF40 and H2Bub1 in siRNF40 CRC cell lines. (A-B). The siRNA-mediated knockdown of RNF40 was verified at the mRNA level using qRT-PCR (A) and on protein level using western blot (B) in three independent experiments 72 h after transfection. Mean ± SEM, Students t test. (C) The knockdown of RNF40 resulted in decreased H2Bub1 levels 72 h after siRNA transfection. (ZIP 7432 kb) [file 13148_2019_698_MOESM1_ESM.zip › Fig.S1B_SW48_RNF40_annotations.tif]

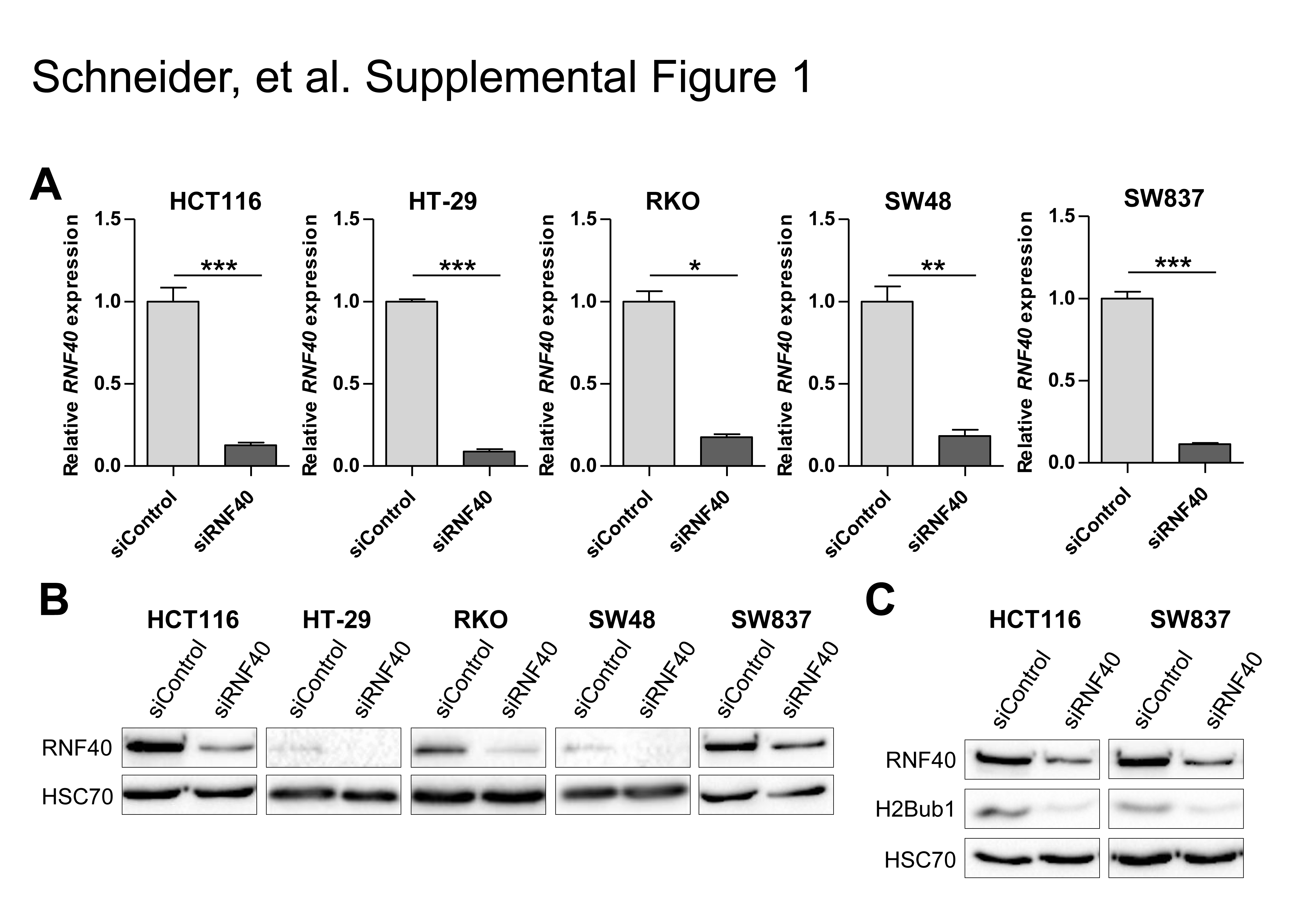

Supplement: Supplementary file 1 — Figure S1. Reduction of RNF40 and H2Bub1 in siRNF40 CRC cell lines. (A-B). The siRNA-mediated knockdown of RNF40 was verified at the mRNA level using qRT-PCR (A) and on protein level using western blot (B) in three independent experiments 72 h after transfection. Mean ± SEM, Students t test. (C) The knockdown of RNF40 resulted in decreased H2Bub1 levels 72 h after siRNA transfection. (ZIP 7432 kb) [file 13148_2019_698_MOESM1_ESM.zip › Schneider et al._Revision_Supp. Figure S1.tif]

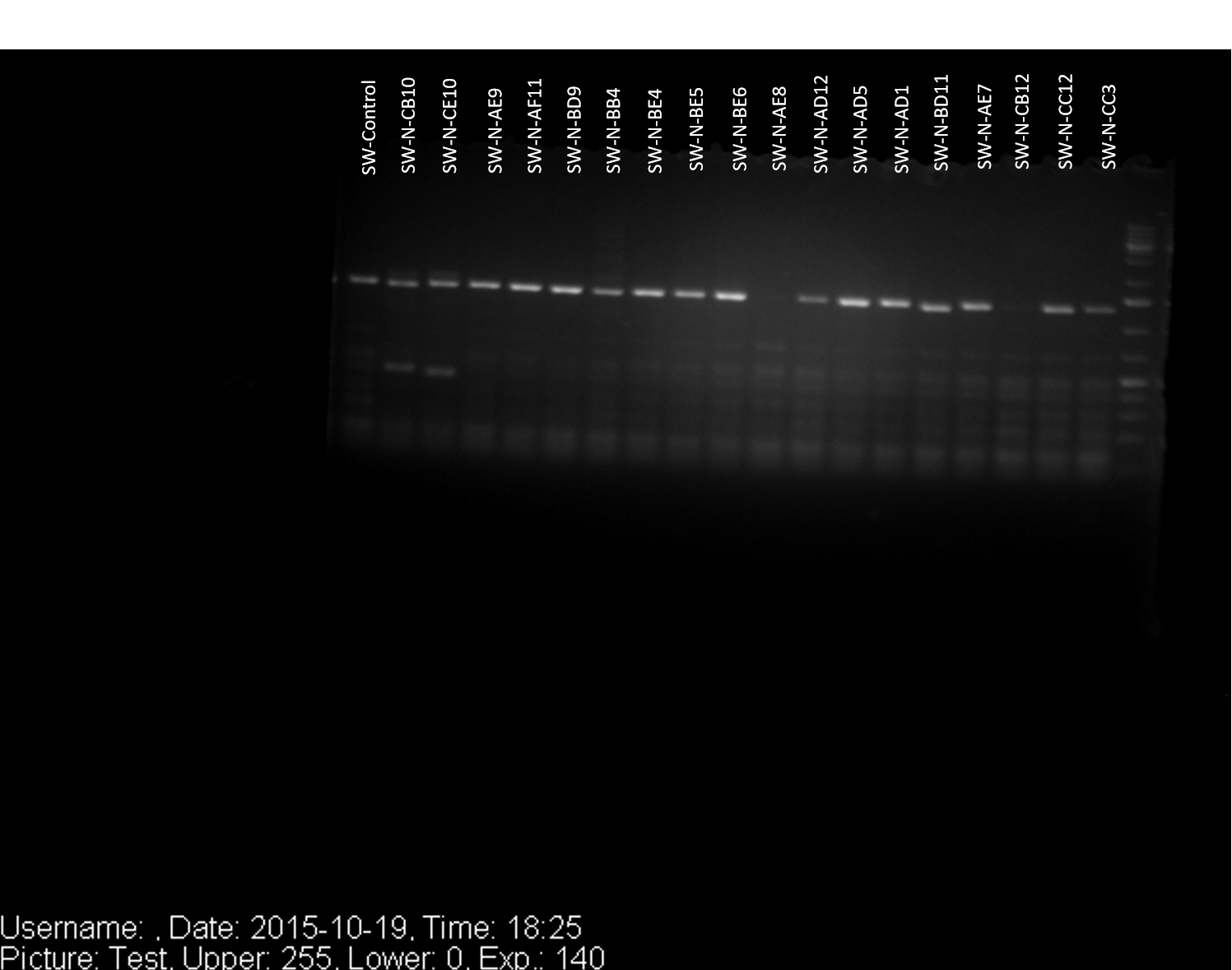

Supplement: Supplementary file 2 — Figure S2. RNF40 knockout is lethal in colorectal cancer cells in vitro. (A) Diagram showing the approach to establish a permanent RNF40 knockout SW480 cells. Two gRNAs were targeted at intron regions before exon 3 and after exon 4 leading to a frameshift and a non-functional protein product. (B) Immunofluorescence assay showing the lack of RNF40 detection (red) when expressing GFP (green) which indicates successful transfection with the Cas9 construct. Scale bar: 10 μM. (C) A scatter plot showing the GFP-positive cells in non-transfected cells and transfected cells. Single cells were picked from the P4 population which is the shows highest GFP expression. (D) PCR amplification product detection on an agarose gel with no clone showing only the expected band (312 bp) upon successful deletion of RNF40. Control: Non transfected triplicate samples (2wA-C) of cells transfected with empty vectors, 24 h A, B, C: triplicates of SW480 cells transiently transfected with Cas9 after 24 h, 120 h A,B,C: triplicates of SW480 cells transiently transfected with Cas9 after 120 h, clones starting with M are MDA-MB-231 RNF40 knockout clones shown as comparison, clone numbers for SW480 (letter of plate, well number). (ZIP 5542 kb) [file 13148_2019_698_MOESM2_ESM.zip › Fig.S2D_CRISPR Cas9 genotyping_1_annotations.tif]

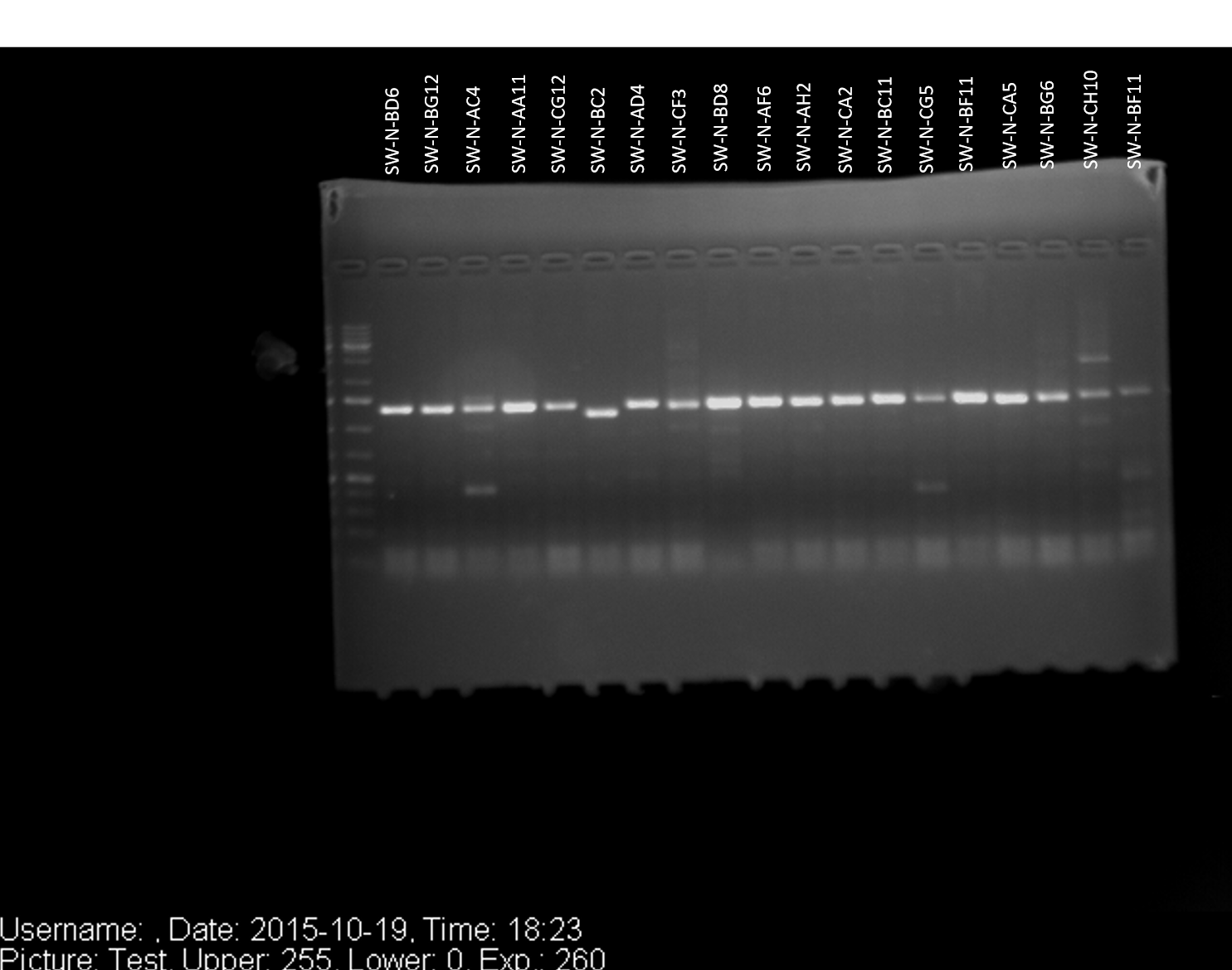

Supplement: Supplementary file 2 — Figure S2. RNF40 knockout is lethal in colorectal cancer cells in vitro. (A) Diagram showing the approach to establish a permanent RNF40 knockout SW480 cells. Two gRNAs were targeted at intron regions before exon 3 and after exon 4 leading to a frameshift and a non-functional protein product. (B) Immunofluorescence assay showing the lack of RNF40 detection (red) when expressing GFP (green) which indicates successful transfection with the Cas9 construct. Scale bar: 10 μM. (C) A scatter plot showing the GFP-positive cells in non-transfected cells and transfected cells. Single cells were picked from the P4 population which is the shows highest GFP expression. (D) PCR amplification product detection on an agarose gel with no clone showing only the expected band (312 bp) upon successful deletion of RNF40. Control: Non transfected triplicate samples (2wA-C) of cells transfected with empty vectors, 24 h A, B, C: triplicates of SW480 cells transiently transfected with Cas9 after 24 h, 120 h A,B,C: triplicates of SW480 cells transiently transfected with Cas9 after 120 h, clones starting with M are MDA-MB-231 RNF40 knockout clones shown as comparison, clone numbers for SW480 (letter of plate, well number). (ZIP 5542 kb) [file 13148_2019_698_MOESM2_ESM.zip › Fig.S2D_CRISPR Cas9 genotyping_2_annotations.tif]

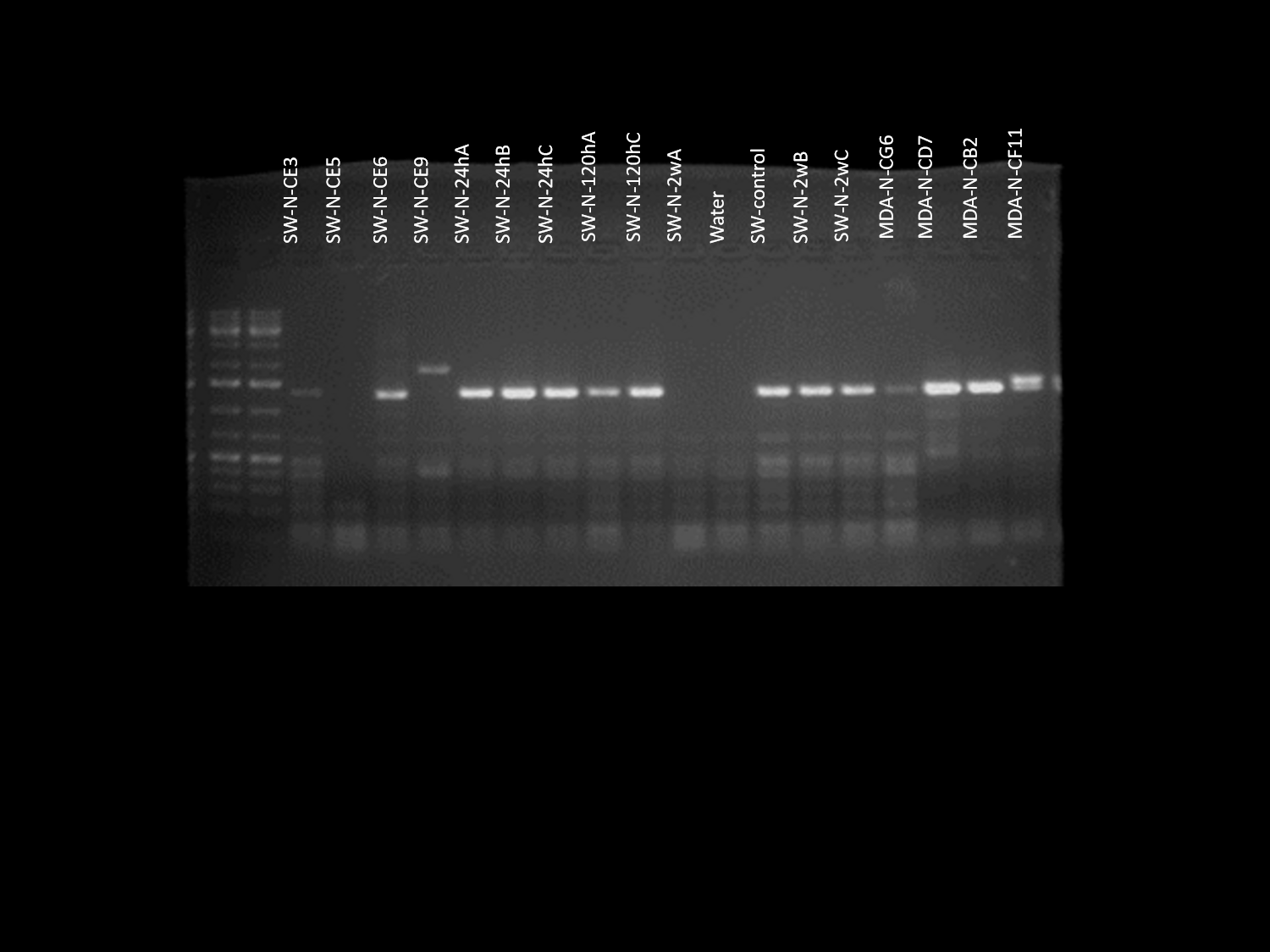

Supplement: Supplementary file 2 — Figure S2. RNF40 knockout is lethal in colorectal cancer cells in vitro. (A) Diagram showing the approach to establish a permanent RNF40 knockout SW480 cells. Two gRNAs were targeted at intron regions before exon 3 and after exon 4 leading to a frameshift and a non-functional protein product. (B) Immunofluorescence assay showing the lack of RNF40 detection (red) when expressing GFP (green) which indicates successful transfection with the Cas9 construct. Scale bar: 10 μM. (C) A scatter plot showing the GFP-positive cells in non-transfected cells and transfected cells. Single cells were picked from the P4 population which is the shows highest GFP expression. (D) PCR amplification product detection on an agarose gel with no clone showing only the expected band (312 bp) upon successful deletion of RNF40. Control: Non transfected triplicate samples (2wA-C) of cells transfected with empty vectors, 24 h A, B, C: triplicates of SW480 cells transiently transfected with Cas9 after 24 h, 120 h A,B,C: triplicates of SW480 cells transiently transfected with Cas9 after 120 h, clones starting with M are MDA-MB-231 RNF40 knockout clones shown as comparison, clone numbers for SW480 (letter of plate, well number). (ZIP 5542 kb) [file 13148_2019_698_MOESM2_ESM.zip › Fig.S2D_CRISPR Cas9 genotyping_3_annotations.tif]

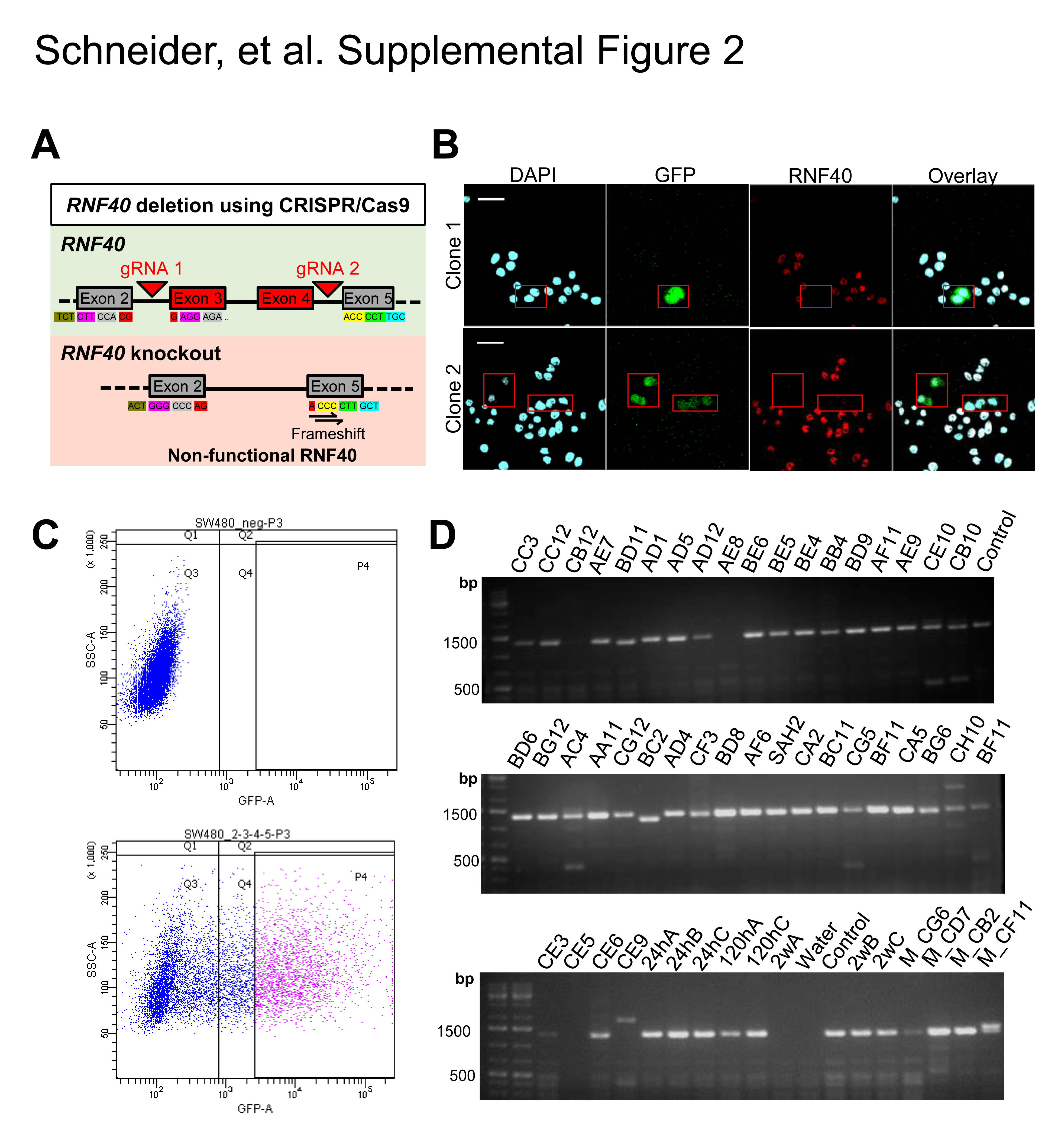

Supplement: Supplementary file 2 — Figure S2. RNF40 knockout is lethal in colorectal cancer cells in vitro. (A) Diagram showing the approach to establish a permanent RNF40 knockout SW480 cells. Two gRNAs were targeted at intron regions before exon 3 and after exon 4 leading to a frameshift and a non-functional protein product. (B) Immunofluorescence assay showing the lack of RNF40 detection (red) when expressing GFP (green) which indicates successful transfection with the Cas9 construct. Scale bar: 10 μM. (C) A scatter plot showing the GFP-positive cells in non-transfected cells and transfected cells. Single cells were picked from the P4 population which is the shows highest GFP expression. (D) PCR amplification product detection on an agarose gel with no clone showing only the expected band (312 bp) upon successful deletion of RNF40. Control: Non transfected triplicate samples (2wA-C) of cells transfected with empty vectors, 24 h A, B, C: triplicates of SW480 cells transiently transfected with Cas9 after 24 h, 120 h A,B,C: triplicates of SW480 cells transiently transfected with Cas9 after 120 h, clones starting with M are MDA-MB-231 RNF40 knockout clones shown as comparison, clone numbers for SW480 (letter of plate, well number). (ZIP 5542 kb) [file 13148_2019_698_MOESM2_ESM.zip › Schneider et al._Revision_Supp. Figure S2.tif]
