# Supplementary material for: The E3 ubiquitin ligase RNF40 suppresses apoptosis in colorectal cancer cells
Source: Clin Epigenetics. 2019 Jul 2;11:98. doi: 10.1186/s13148-019-0698-x (PMC6604314; doi:10.1186/s13148-019-0698-x)
Supplement: Supplementary file 3 — Tables S1–S3, Supplemental tables. (DOCX 16 kb) [file 13148_2019_698_MOESM3_ESM.docx]

Additional file 3

Table S1: gRNA sequences used in this study to deplete *RNF40*.

| gRNA | Sequence (5’-3’) |
| --- | --- |
| gRNA 1 sense | CACCGACCGCTAACAGAATCTAAGG |
| gRNA 1 antisense | CACCGACCGCTAACAGAATCTAAGG |
| gRNA 2 sense | CACCGTGTTGCACTTGCTATAAGGG |
| gRNA 2 antisense | AAACCCCTTATAGCAAGTGCAACAC |

Table S2: qRT-PCR primers used in this study.

| Gene |  | Sequence (5’-3’) |
| --- | --- | --- |
| *18sRNA* | F | AACTGAGGCCATGATTAAGA |
|  | R | GGAACTACGACGGTATCTGA |
| *RNF40* | F | AGTACAAGGCGCGG |
|  | R | GAAGCAGAAAACGTGGAAGC |
| *BCL2L11* | F | TGATTCTTGCAGCCACCCTG |
|  | R | GGGGAACAAGGGCCAAGAAA |
| *BAX* | F | CCCGAGAGGTCTTTTTCCGAG |
|  | R | CCAGCCCATGATGGTTCTGAT |
| *MCL1* | F | ATCTCTCGGTACCTTCGGGAGC |
|  | R | CCTGATGCCACCTTCTAGGTCC |
| *BIRC5* | F | ACGACCCCATAGAGGAACAT |
|  | R | TCCGCAGTTTCCTCAAATTC |

Table S3: Primary antibodies used in this study.

| Antibody | Host | Cat. no. | Company |
| --- | --- | --- | --- |
| RNF40 | Rabbit | ab191309 | Abcam |
| H2Bub1 | Rabbit | 5546 | Cell Signaling |
| H2B | Rabbit | ab1790 | Abcam |
| PARP | Rabbit | 9542 | Cell Signaling |
| BIM | Rabbit | 2933 | Cell Signaling |
